# Photodynamic Therapy: A Compendium of Latest Reviews

**DOI:** 10.3390/cancers13174447

**Published:** 2021-09-03

**Authors:** José Francisco Algorri, Mario Ochoa, Pablo Roldán-Varona, Luís Rodríguez-Cobo, José Miguel López-Higuera

**Affiliations:** 1 Photonics Engineering Group, University of Cantabria, 39005 Santander, Spain; mario.ochoa@unican.es (M.O.); pablo.roldan@unican.es (P.R.-V.); lopezhjm@unican.es (J.M.L.-H.); 2 CIBER-bbn, Institute of Health Carlos III, 28029 Madrid, Spain; luis.rodriguez@unican.es; 3 Instituto de Investigación Sanitaria Valdecilla (IDIVAL), 39011 Santander, Spain

**Keywords:** photodynamic therapy, cancer, overview, review, meta-analysis

## Abstract

**Simple Summary:**

In recent years, numerous general and specific reviews about photodynamic therapy and related subtopics have been published. The diversity of reviews is broad, including many different complex topics and disciplines. In this work, we aim to summarize and compile the latest reviews covering the whole photodynamic therapy field. Specifically, the most important reviews of the last two decades are described. Additionally, some of the key issues and main conclusions detected in these reviews are included. We hope this work will serve as a guide and starting point for researchers to delve further into specific topics and formulate research questions.

**Abstract:**

Photodynamic therapy (PDT) is a promising therapy against cancer. Even though it has been investigated for more than 100 years, scientific publications have grown exponentially in the last two decades. For this reason, we present a brief compendium of reviews of the last two decades classified under different topics, namely, overviews, reviews about specific cancers, and meta-analyses of photosensitisers, PDT mechanisms, dosimetry, and light sources. The key issues and main conclusions are summarized, including ways and means to improve therapy and outcomes. Due to the broad scope of this work and it being the first time that a compendium of the latest reviews has been performed for PDT, it may be of interest to a wide audience.

## 1. Introduction

According to the Global Cancer Observatory (GCO), cancer is a leading cause of death worldwide, accounting for nearly 10 million deaths in 2020 [1]. The established standard treatment strategies (surgery, radiotherapy, and chemotherapy) have demonstrated reasonable success for specific cancers in recent years. Despite this, some issues such as resistant cancer cells, recurrence, and metastases have not been overcome. For this reason, new technologies are essential for future cancer treatments.

**Figure 1:**
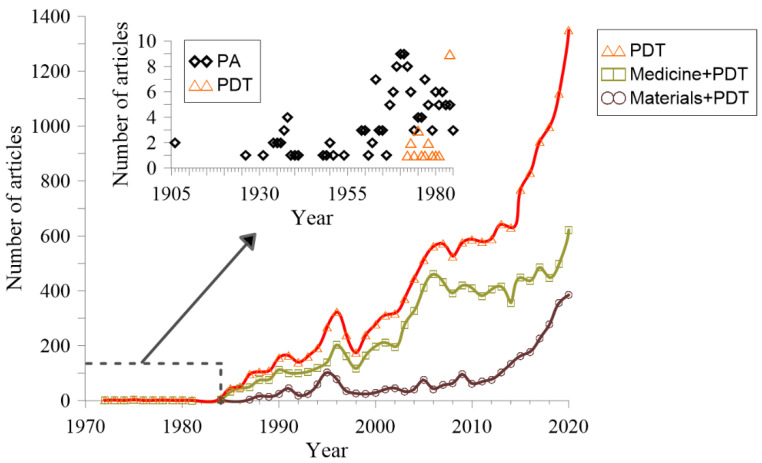
Number of articles per year as a result of the search in the Scopus database (Elsevier). The search was restricted to only the titles of the articles (not restricted to review articles). Queries for each line were: TITLE (“photodynamic therapy”) red line; TITLE (“photodynamic therapy”) AND SUBJAREA (MEDI) green line; TITLE (“photodynamic therapy”) AND SUBJAREA (MATE) purple line. Note that the subject area is overlapped in some journals (materials and medicine are selected because they are more differentiated). Inset shows the difference in articles between Photodynamic Action (PA, dark symbol) and Photodynamic Therapy (red symbol).

A non-conventional therapeutic modality for solid tumours, which offers advantages over standard treatments, is photodynamic therapy (PDT) [2], a light-based technology. Light has been used in medical treatments for over a millennium. It was used in ancient Egypt, India, and China to treat skin diseases, such as psoriasis, vitiligo, and cancer, as well as rickets and even psychosis [3]. Despite this, sunlight as a medical treatment was not widely used until the eighteenth century. The Nobel Prize awarded to Niels Finsen acknowledged phototherapy in 1903. He was granted this prize for treating cutaneous tuberculosis by using ultraviolet (UV) light. Simultaneously, J. Prime discovered that patients with epilepsy treated orally with eosin were sensitive to the sun on exposed areas of the skin, causing dermatitis [4]. Thanks to this discovery, H. Tappeiner and A. Jesionek employed a topical version of eosin along with a white light source to treat skin tumours [5]. After that, the term ‘‘photodynamic action’’ was coined by H. Tappeiner and A. Jodlbauer when they discovered the oxygen requirement in photosensitization reactions [6]. After this pioneering work, little research was performed until 1972 (few research articles per year according to Scopus; see Figure 1). In the same year, Diamond et al. achieved a reduction of tumour growth for some days [7], concluding that photodynamic action (renamed as photodynamic therapy, PDT) could be used as a new approach to treat this disease. In the inset of Figure 1, it is possible to see the tendency of research articles under these two terms. In 1975, the first significant milestone in PDT occurred at the Roswell Park Cancer Institute in Buffalo. T. J. Dougherty et al. reported the first successful complete tumour cure in animal specimens by using PDT [8], heralding a new era for this technique. The first PDT clinical trials date back to the late 1970s [9]. In that study, the effects of light and hematoporphyrin derivative (HpD) in five patients with bladder cancer were studied. Later in 1978, Dougherty reported the first large series of patients successfully treated with PDT [10]. Several types of tumours were analysed, and all of them demonstrated a response to the treatment. Since then, there have been more than 283 trials for PDT (44 currently active) [11].

Photodynamic therapy is based on three main elements: a photosensitizer (PS), light, and molecular oxygen. The typical process begins with the PS uptake, either topically or systemically injected. In most cases, after some time, the PS reaches a maximum concentration within the vasculature and in some cases also in the tumour. It has to be noted that one clinically approved PS works in a different way; Padeliporfin (Tookad Soluble, WST11) does not accumulate either in cells or in the vascular endothelium but only creates a complex with albumin and acts as a photocatalyst according to the Type I mechanism. Some mechanisms of selective PS retention include a high proliferative rate of neoplastic cells, lack of or poor lymphatic drainage, binding to low-density lipoproteins, increased vascular permeability, abnormal structure of tumour stroma (with an increased amount of collagen that binds to porphyrins), and infiltration of macrophages in the tumour (that trap hydrophobic PS) [12,13]. When it reaches the maximum PS concentration at the tumour, with respect to the healthy tissue, it is time for light to be applied. The PS usually has maximum peaks of absorption at some specific light wavelengths. When the light interacts with the PS, it can transfer its excited-state energy into either tissue substrate or surrounding oxygen. Thanks to this energy transfer, reactive oxygen species (ROS) are produced. Two types of reactions can occur, and they generate superoxide anion radicals and reactive singlet oxygen molecules, which kill tumour cells by direct cell death (necrosis or apoptosis), vascular damage (leading to tissue ischemia), immune modulation, or a combination of these. Some advantages of PDT over standard treatments are its localization (thanks to direct light application and PS concentration at the tumour), cytotoxic mechanisms (no damage to DNA and connective tissue structures) [14], possibility for repeated treatment, inhibition of drug resistance pathways [15,16], and activation of immunological responses [17], among others. The efficacy of these mechanisms depends on many factors, such as the type of PS, cell, overall light dose, and tumour oxygenation status, among others. These advantageous characteristics make PDT a feasible option to be used alone or as complementary (before or after) to current standard therapies. They forecast great potential for PDT in multi-therapy settings. It is of paramount importance for patients with an inadequate or incomplete response to neoadjuvant therapies (NATs) or in cases where NAT and surgery are not possible. There may even be circumstances in which PDT could be an acceptable alternative to surgery or radiotherapy. Furthermore, in the clinical field, the repeatability and “commutativity” of treatments are much-appreciated characteristics, and PDT-based ones can offer both.

In the last two decades, an exponential increase in research has been performed to tackle the PDT challenges (see Figure 1). Figure 1 depicts the number of articles per year since 1905. As mentioned before, the term “Photodynamic Therapy” was not used until 1972 [7]. Thus, the main graph represents the articles found for PDT since this year. Previous years can be found in the inset, including PDT and Photodynamic Action (PA). Furthermore, in the main graph, two additional lines represent the articles for different subjects (Medicine and Materials).

The exponential growth this field is currently undergoing is clear, with a significant contribution to the growing body of literature from the great advancement in material science related to PDT (thanks to novel nanotechnologies). According to the Scopus database, the number of review publications in the last two decades is 353 (words in the title or keywords by the author “photodynamic therapy” and “cancer or tumor or tumour”: TITLE(photodynamic AND therapy) AND TITLE(cancer OR tumor OR tumour) AND PUBYEAR > 2000 OR AUTHKEY(photodynamic AND therapy AND cancer) OR AUTHKEY(photodynamic AND therapy) OR AUTHKEY(photodynamic AND therapy AND tumor) OR AUTHKEY (photodynamic AND therapy AND tumour) AND (LIMIT-TO (DOCTYPE, “re”)). Due to the large and diverse body of literature, we have selected the most important reviews of the last two decades, in which some of the most relevant articles are summarised. For this, the search is divided into two steps: first, we focus on key topics (detailed below); second, we select the most relevant articles (at least 50 references or highly positioned by search engines). We will also present the key issues and conclusions detected in some of these reviews, including ways and means to improve therapy and outcome. We hope this contribution will be of interest to a broad audience, from bioengineers to clinical oncologists. We do not aim to perform an exhaustive critical review of reviews of the multidisciplinary topics included in this work (attempting to cover the whole PDT field). However, due to the diverse and large body of literature on PDT, we believe that a guide, compilation, and summary of the key messages and conclusions of the latest reviews are needed. Detailed information to deepen understanding about specific topics can be found in the cited articles. Accordingly, a substantial part of the information presented in this contribution is extracted or adapted from the selected reviews.

The following section describes a selection of the latest reviews of PDT. The section is subdivided into groups according to some key topics: overviews, specific cancers and clinical trials, PS, PDT mechanisms, immune response activation, dosimetry, and light sources. Then, we summarize and conclude with the major findings of this review, including some identified suggestions for further research.

## 2. Latest Reviews of PDT

Photodynamic therapy has been extensively studied after its resurgence in the 1970s. The multidisciplinary nature of PDT (medicine, chemistry, biochemistry, materials science, and engineering) makes it a complex and broad field, in which hundreds of publications per year can be found (see Figure 1). Research is typically focused only on specific topics: PS, light sources, dosimetry, the PDT effect (e.g., direct ROS effects, vascular damage, and immune response), cell death pathways (e.g., apoptosis, paraptosis, necrosis and autophagy), or general clinical trials. General overviews, which include many of these subtopics, can also be found, which are advantageous for opening pathways towards enhancing PDT outcomes. Hence, the following section summarizes this kind of review, followed by sections dealing with the reviews on specific subtopics.

### 2.1. Overviews

For basic principles and some history of PDT, including clinical reports, the reader is referred to the broad overview by Mcdonald and Dougherty from 2001 [18]. In the same year, the history of photodetection and PDT was reviewed comprehensively by Ackroyd et al. [3]. The latter traced the origins and development of PDT from antiquity to the present, describing how PDT was first implemented and some history of the most common PSs. Furthermore, a summary of the clinical trials of the 1990s can be found (organized per body region: oesophagus, skin, brain, etc.). In 2002, a review about general principles of PDT was published [19]. Another review, mainly about history but focused on the last century, was published in 2003 [13]. The same year, Dolmans et al. published a general overview [20], being the most cited review of the last two decades (4339 citations). Another highly cited review was published the following year (1314 citations) [21]. In the latter reference, Brown et al. presented an overview of PDT and a perspective considering the novelty of PS in this age. After that, in 2008, Wilson and Patterson wrote another overview that outlined the clinical status at that time, with an emphasis on the contributions of physics, biophysics, and technology (biological targets, light propagation in tissue, PDT technologies, and dosimetry) and ending with a discussion on the challenges remaining in optimisation and adoption by the medical sector [22]. An updated overview was written by Agostinis et al. in 2011 [23]. After a brief description of some essential components of PDT, Agostinis et al. focused on some PDT cell death mechanisms, such as vascular damage or immune response. To conclude, the review summarized some clinical trials on the brain, skin, head, neck, and tumours of the digestive and urinary systems. In addition, an exciting discussion about the current and future direction of PDT can be found. It would take until 2017 to see another excellent overview produced, written by Straten et al. [24]. This review provided a broad overview of oncologic PDT applied in a clinic from 2006 to 2016. Furthermore, it included the basic principles of PDT, some preclinical studies, limitations, and a discussion on possible directions for PDT in the following years. Finally, the most updated overview can be found in Refs. [25,26,27,28] (2019), Ref. [29] (2020), and Ref. [30] (2021).

The previous reviews coincided with the need to face some particular issues before integration into mainstream treatment. They have been selected and arranged in line with the subsequent sections. For example, as stated in [24], one crucial issue is the lack of randomized controlled clinical trials. These are usually trials of small size (with different treatment protocols), in which PDT is not used independently or is not directly compared with standard treatments (see Section 2.2). In spite of the lack of randomized controlled clinical trials, similar outcomes have been demonstrated for small lesions (early detection) for cases in which PDT has been compared to surgery (e.g., in the field of head and neck cancer).

In contrast, surgery proves more effective than PDT for cancers with bigger or deeper lesions (lung, pancreas, and biliary tract cancers that are detected at a later stage). In chemotherapy or radiotherapy, PDT has proved to be an effective and safe alternative with lower morbidity. However, it has to be noted that established treatments are commonly used in most hospitals. In contrast, PDT uses specific equipment not usually available in the clinical setting (even though it is less expensive) [24]. Another issue commented on in the latter reference is that almost one-third of the recent clinical trials still use first-generation PS. Therefore, the future direction of study has to focus on third-generation PS (commented in Section 2.3) rather than trying to establish a firm position for well-established PS [24]. Another issue noted in [25] is that PDT selectivity is not absolute, and some damage can occur to the surrounding tissue. Hence, it is relevant for understanding the molecular mechanisms involved in resistance, drug delivery, and specific targeting of tumours to develop advanced technologies to enhance specificity and reduce resistance to PDT treatment (see Section 2.4). In this regard, tumour hypoxia is an obstacle for many cancer therapies [24]. Another exciting aspect being studied is activating an immune response following tumour cell death caused by PDT (see Section 2.4). Several reviews have been published in recent years due to the great number of articles with preclinical results, including data from clinical trials. As commented on in [24], preclinical research has shown the immunological events after PDT and their effect on treatment outcome. It also has to be noted that cancer vaccines have been proposed (based on PDT-treated tumour cells), proving a reduction in tumour size or stopping tumour growth [24].

Finally, it is well-known that dosimetry is of paramount importance and has a significant impact on PDT efficacy. Real-time dosimetry providing optimized and adapted treatments has shown great potential in some instances; however, there are still several challenges (see Section 2.5). In the latter section, readers can also find the latest reviews of light sources. In this case, reviews can be generic or consist of specific topics.

### 2.2. Specific Cancers and Meta-Analyses

Besides the aforementioned topical reviews, other diverse reviews (including systematic reviews) focus on specific topics concerning PDT. Most of the peer-reviewed articles consist of particular cancer treatments and meta-analyses of clinical trials. Table 1 summarises reviews of the last decade (searched in Scopus database) for PDT applied to some of the most researched types of cancers (gastrointestinal, skin, lung, prostate, head and neck, breast, brain, and bladder). The search was performed in the Scopus database with the keywords in the title: photodynamic therapy; cancer; and cancer type. Additionally, the search was limited to review-type documents since 2010.

In the following subsections, we summarize some of the main conclusions and critical issues detected in the reviews of Table 1. The presented information is extracted or adapted from the selected reviews, as the authors do not intend to make a critical revision of them but a summary of relevant conclusions. For more details, interested readers can check the referenced articles.

#### 2.2.1. Gastrointestinal Cancer

Gastrointestinal cancer encompasses several types of cancers depending on the tumour site; the most common are cholangiocarcinoma (vile ducts), colon, pancreas, and liver. For cholangiocarcinoma, authors of Ref. [31] concluded that results are best if treatment is performed within the first month after diagnosis and Karnofsky status is more than 30. In Reference [32], it was concluded that palliative PDT in bile duct cancer improves survival but increases the risk of cholangitis and liver abscess; the phototoxicity of the skin is of ancillary importance. Additionally, PDT should be confined to patients without distant metastases and patients with a tumour extent of ≤3 cm in diameter [32]. A complete review is [38], which compared outcomes with PDT therapy and biliary stenting vs. biliary stenting only, concluding that PDT seems to be significantly superior to biliary stenting alone. For this reason, the recommendation was to use PDT as an adjuvant therapy to biliary stenting in these patients [38]. For the colon, a state-of-the-art in clinical research can be found in [37] and in preclinical research in [35]. The following years brought reviews focused on targeted therapies for colon PDT [39], immunotherapy and colon PDT [40], or even the combination of PDT with cannabidiol [42]. These reviews validated the combination of PDT with alternative treatments. Furthermore, they highlighted that the combination of various treatments (including targeted, immunotherapy, or cannabidiol) might be a justified strategy for colorectal cancer treatment. PDT has proved a possible option for minimally invasive treatment for the pancreas, but it is still at an early stage of development [36]. In Ref. [43], current clinical studies and the advantages of nanoparticles (NPs) in boosting therapeutic efficacy were summarized. The main conclusion was that present studies have demonstrated the potential of NPs-based PDT for the pancreas, and further studies are warranted to optimize NPs design and investigate their long-term safety and efficacy [43]. The existing clinical studies and continuing phase II/III studies of PDT in pancreas form a good basis for developing NPs-based PDT against pancreas cancer [43]. Finally, clinical outcomes reveal that PDT can be considered a promising treatment modality for all liver cancers (including metastases) to improve the quality and extension of a patient’s life [41]. On the contrary, PDT studies of humans require better light-delivery methods [41].

#### 2.2.2. Skin Cancer

Skin cancer includes all types of cancer that start in the skin. Skin colour and exposure to both natural and artificial ultraviolet radiation (sunlight, tanning beds, etc.) can increase the risk of developing non-melanoma skin cancer and actinic keratosis. In [44], the main conclusion was that for actinic keratosis (AK), there was no clear evidence of the superiority of PDT concerning other treatments. For Bowen’s disease (BD), better outcomes with PDT were suggested compared with cryotherapy or fluorouracil. Reference [45] added that the use of topical PDT could be an effective therapy for BD, equivalent to other therapies such as cryotherapy, and even superior to topical 5-FU. Its cosmetic outcome is superior to standard treatment. Other common skin cancers are basal cell carcinoma (BCC), which forms in the basal cells, and squamous cell carcinoma (SCC), which originates in the squamous cells [44]. For BCC, PDT may result in similar lesion response rates to surgery or cryotherapy but with better cosmetic outcomes. Authors of Reference [45] remarked that topical MAL-PDT is effective in nodular BCC, but the efficacy is lower than surgery. For this reason, the use of one or the other must be assessed according to the expected results of the surgery. In [54], a systematic review and meta-analysis (11 articles with seven randomized controlled trials, 1339 patients, 1568 lesions) and one retrospective study, 108 lesions) were carried out to compare the benefits and tolerance of MAL-PDT compared with other traditional modalities for the treatment of BCC. The main conclusion was that MAL-PDT could not be the best option as a first-line treatment for BCC. Despite this, the cosmetic outcome was excellent in some cases. In [46], a systemic search was carried out that focused on the main clinical studies using mTHPC-PDT on non-melanoma skin cancer in humans, concluding that despite mTHPC-PDT being described in the literature as a promising and interesting therapeutic option, especially for multiple non-melanoma skin cancers, there is not enough evidence to assess this. Some studies indicate that mTHPC-PDT is a good option not as the first line of treatment but as a last resort therapy [46]. In [45], it was also noted that primary SCC lesions of the skin are amenable to PDT treatment. Despite this, PDT was not recommended to be used independently for SCC lesions with a potential risk of spreading (due to the limited penetration depth in local treatments). For individuals at risk of lymphatic spread, surgical excision of the primary and nodal drainage region remains the standard of care [45]. For a remarkable recent update, readers can check the work of Hamblin et al. [96] (95 citations). Finally, a combined strategy was studied in [47], concluding that depending on the characteristics and the type of tumour, PDT can be applied in combination with immunomodulatory (Imiquimod) and chemotherapeutic (5-fluorouracil, methotrexate, diclofenac, or ingenol mebutate) agents, inhibitors of some molecules implicated in the carcinogenic process (COX2 or MAPK), surgical techniques, or even radiotherapy [47].

#### 2.2.3. Lung Cancer

Lung cancer consists of a group of diseases resulting from the malignant growth of cells of the respiratory tract, particularly lung tissue, and is one of the most common types of cancer worldwide. Lung cancer usually originates from epithelial cells and can lead to metastasis and infiltration of other body tissues. Lung cancer excludes those neoplasms that metastasize to the lung from tumours in other parts of the body. PDT can be used for early-stage lung cancer, advanced lung cancer with airway obstruction, or preoperative procedures. The mechanisms behind this can be found in Ref. [58]. In Ref. [56], all types of lung cancer were reviewed, concluding that PDT behaves differently for each of them. For example, for centrally located early-stage lung cancer, a successful PDT requires a tumour located at the mucosa and submucosa, because tumour invasion as estimated by surface diameter is not always accurate (tumours ≤1.0 cm may show extracartilaginous invasion). For this reason, it is necessary to improve the estimation of the tumour margin and its depth of bronchial wall invasion. PDT has been used as a palliative treatment for inoperable patients with advanced lung cancer with airway obstruction. In Ref. [56], a summary of clinical trials can be found. On the other hand, the authors of Ref. [57] focused on PDT for central-type early-stage lung cancer, concluding that to obtain a complete response by PDT, the selection of indicators is critical, including the extent of the tumour on the surface of the bronchial wall and the depth of the invasion in the bronchial wall. Similar to the previous reference, the conclusion is that PDT has a favourable cure rate for tumours less than 1 cm in diameter. Even by using NPe6 PS, large tumours can be treated [57]. It has to be noted that similar conclusions can be found in Ref. [59].

In the case of advanced lung cancer with airway obstruction, endobronchial PDT is a minimally invasive technique that can be used to reduce the obstruction [59]. As noted in [60], PDT can decrease airway obstruction and tumour stenosis for this patient population, often leading to better respiratory function. Furthermore, the use of PDT could solve acute hemoptysis and poststenotic pneumonia. An important summary was reported in [62], including all the clinical studies from 2006–2010, with a total of 493 patients using PDT with different PSs, in the treatment of early-stage (*n* = 370) and advanced (*n* = 123) lung cancer. It is worth noting the higher rates of complete response for early-stage disease, ranging from 72% to 100% between different studies. On the other hand, when the disease is advanced, the considered cases are local control and partial response, ranging from 78 to 100%, respectively [62].

Finally, some reviews have covered specific topics in recent years, e.g., nanoparticle photosensitizer drug delivery uptake systems [63] or a combination of PDT and chemotherapy [65]. Additionally, an update of clinical trials can be found in [64].

#### 2.2.4. Prostate Cancer

**Figure 2:**
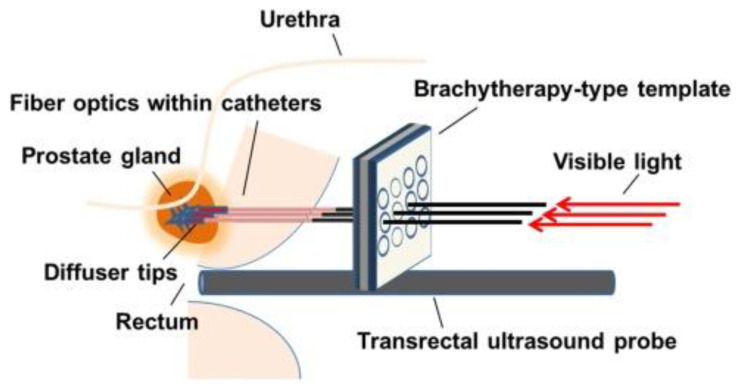
A generalized diagram depicting the use of a brachytherapy-type template and transrectal ultrasound to precisely guide catheters containing fibre optics to the prostate gland for PDT. Reproduced with permission from M. Osuchowski, Photodiagnosis Photodyn. Ther. 2021 [73].

Prostate cancer is also a common type of cancer. In many cases, prostate cancer grows slowly and is confined to the prostate gland, where it may not cause severe damage. Despite this, whereas some types of prostate cancer have a low growth (needing minimal or even no treatment), other types are more aggressive and spread quickly. The most common PDT technique used in prostate cancer is interstitial PDT (see Figure 2). PDT for prostate cancer has been extensively studied since the late 2000s (by pioneering research groups from Lund University, University of Pennsylvania School of Medicine, and Ontario Cancer Institute). In [68], a review of PDT in prostate with palladium-based PS (WST-09 and WST-11), the authors remarked that vascular-targeted photodynamic therapy (VTP) with good tolerability and efficacy is a good option as an intermediate treatment for local low-risk disease, midway between active surveillance and radical therapy. The same was concluded in [69], in which several PS were reviewed: aminolevulinic acid (5-ALA), verteporfin, mTHPC (Foscan), motexafin lutetium (MLu), and TOOKAD. Despite this, the long-term benefit compared to traditional treatments has to be studied. In a more recent review, the results were that in phase II studies, VTP showed a rate of 80% negative biopsies at six months, with good clinical tolerance [70]. A European phase III randomized prospective study, which compares VTP to active surveillance, showed a lower proportion of progression and a more significant duration before progression for VTP [70]. The adverse events are primarily moderate and transient. Patients’ quality of life is preserved, with a moderate impact on erectile and urinary function [70]. However, in Ref. [72] it is noted that despite meta-analysis results show good effectivity, there are not only low levels of side effect rates but also insignificant effects on both urinary and erectile function. More high-quality randomized controlled trials are needed to evaluate the comparative efficacy, safety, and functional outcomes of PDT for patients with prostate cancer [72]. Finally, two reviews have been reported this year. In [73], an exciting discussion of active treatment vs. active surveillance for patients with low-grade prostate cancer included critical points from clinical trials using PDT for prostate cancer with several PSs and recently developed methodologies for photodynamic prostate cancer treatment are in the experimental stage [73]. The article highlighted that despite there have been significant advancements in the last decade for early focal treatment of low-risk localized prostate cancer, a single well-defined PDT dose metric is yet to be determined, and that is why it is difficult to predict its impact [73]. Authors of Ref. [74] performed a comparative study of several minimally invasive therapies: cryoablation, high-intensity focused ultrasound, irreversible electroporation, and vascular-targeted PDT for prostate cancer. This was the first study to include a quantitative evaluation of the clinical outcomes among different ablative technologies. Although this meta-analysis showed that these techniques are promising therapies for prostate cancer patients with similar clinical outcomes, the heterogeneous body of focally treated patients (including both low-risk and intermediate-risk patients, multiple tissue-preserving strategies, several energy modalities, and diverse outcomes) precludes drawing conclusions on the ideal energy applied and optimal success rates [74].

#### 2.2.5. Head and Neck Cancer

Head and neck cancers usually begin within the squamous cells that line the mucosal surfaces of the pinnacle and neck (e.g., inside the mouth, throat, and voice box). These cancers are noted as epithelial cell carcinomas of the head and neck. Head and neck cancers are also considered those beginning within the salivary glands, sinuses, or muscles or nerves within the head and neck, but these are less common than the previous ones [97]. PDT for head and neck cancer has been extensively studied. In the last decade, the first review was about the management of pre-malignant head and neck mucosal dysplasia and microinvasive carcinoma [75]. The main conclusions were that PDT has several advantages, including treating a broad surface area superficially without the risk of developing fibrotic and functional debilitating complications [75]. Moreover, the ability to repeat treatments without cumulative toxicities could be critical to establish PDT as first-line therapy. Reference [76] focused on reviewing 5-Aminolevulinic acid-mediated PDT for oral cancers. It was concluded that topical ALA-PDT using either a light-emitting diode or laser light is very effective for oral verrucous hyperplasia and oral erythroleukoplakia lesions but is relatively less effective for oral leukoplakia lesions [76]. Additionally, [78] concluded that PDT is effective in the overall management of oral premalignant lesions. In Ref. [77], the authors focused on mTHPC-mediated PDT of squamous cell carcinoma. The main conclusions were that there are insufficient studies for adequate assessment of the efficacy for curative intent, despite this palliative treatment with PDT increasing the quality of life in otherwise untreatable patients [77]. In a more recent review, authors also stated that very little randomized data are available [81], but that current data indicate that for superficial lesions of depth less than 5 mm and early-stage lesions, PDT offers an alternative option to surgery or radiation for oral cavity lesions and laryngeal lesions, which may preserve tissue and lead to better functional outcomes. Additionally, the authors of Ref. [80] stated that PDT is a very accurate and effective therapy in the early stages and can significantly affect surgical outcomes in cancerous patients. Recent progress in the field of PDT focuses on the development and clinical application of new photosensitizing agents, photochemical internalization, and photoimmunotherapy [82].

#### 2.2.6. Breast Cancer

According to the World Health Organisation, breast cancer is the most common female cancer, representing one in four cancers diagnosed among women globally, with more than 2 million new cases diagnosed in 2020. Despite the established standard strategies (surgery, radiotherapy, and chemotherapy) having reasonable success for certain cancers, resistant cancer cells, recurrence, and metastases remain common. For these cases, PDT has been proposed as an alternative therapy. Much work has been done in vitro; for example, in [98], combinations of various therapeutic modalities and tumour-targeted strategies were reviewed. The discovery of more effective cancer photochemotherapy agents is crucial to developing novel receptor-targeting approaches [98]. Another advantage of PDT is that the unique mechanism of photodynamic treatment on a tumour and its microenvironment could inhibit drug resistance pathways and re-sensitize resistant cells to standard therapies [15]. The latter reference summarized the role of membrane ABC efflux transporters in therapeutic outcomes and highlighted research findings related to PDT and its applications for breast cancer with multidrug resistance phenotype [15]. In [83], treatment strategies for breast cancer, with particular attention to photodynamic therapy and natural product-based treatments, were reviewed. The main conclusion was that natural products may be potential candidates as therapeutic agents along with PDT for controlling breast and related cancers by regulating cell signalling, cell cycle alteration, and apoptotic or even autophagic cell death. In 2017, PDT was proposed for primary breast cancer [84], because as it was noted, at that time PDT had been used only as a trial treatment of cutaneous metastasis in patients with advanced disease. The same authors demonstrated the use of I-PDT for breast cancer last year [99]. Another recent review was focused on metastatic intradermal metastatic breast cancer [85]. The main conclusion was that PDT with sodium sinoporphyrin shows great promise to inhibit the growth of both the tumour itself and its metastases. Finally, the most recent review can be found in [87], in which readers can find methods to increase the selectivity and bioavailability of photosensitizers, a complete compilation of in vitro PDT studies, and a perspective on the prospects for breast cancer PDT.

#### 2.2.7. Brain Cancer

A brain tumour is an uncontrolled growth of cells derived from brain components (primary tumours) or tumour cells located in other areas of the body (metastasis). Tumours can be benign or malignant, depending on how fast they grow and whether they can be resected or cured by neurosurgical treatment. Unlike tumours of other tissues, the distinction between benign and malignant manifestations is not so clear; for example, some benign lesions can infiltrate entire regions with malignant clinical behaviour. On the other hand, malignant neoplasms metastasize, which is an exceptional event. Metastases to the central nervous system come, in order of frequency, from the lung, breast, skin (melanoma), kidney, and gastrointestinal system and tend to grow between the union of the cortex and the white matter. Brain cancer has a poor outcome due to the difficulty of precisely resecting the tumour (no visible borders and marginal zones).

**Figure 3:**
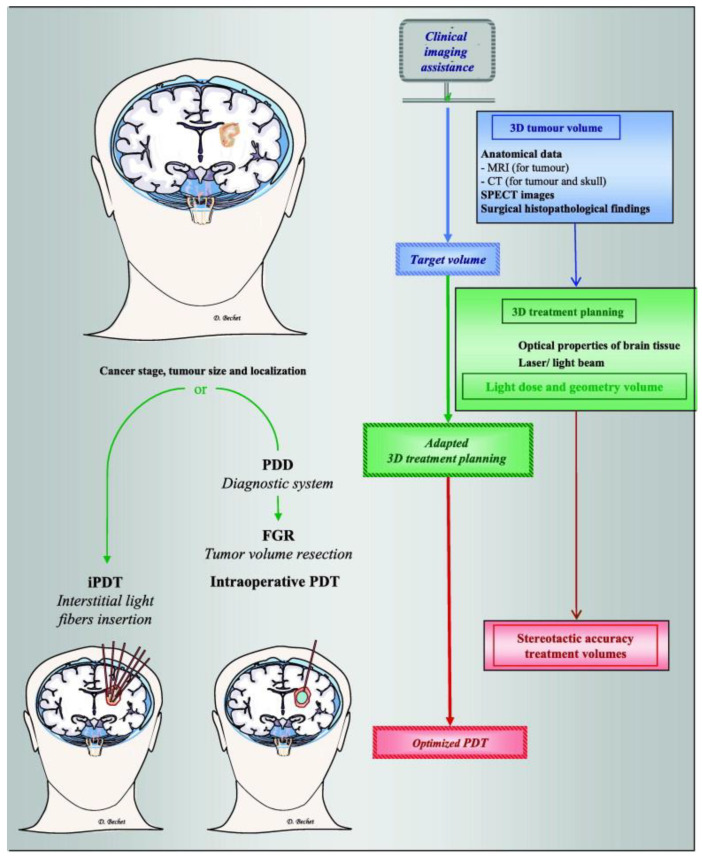
Clinical imaging assistance as an essential step in therapeutic planning. Left side: Two strategies suggested depending on the stage, size, and localization of the tumour. An interstitial PDT (iPDT) or, after a minimally invasive craniotomy, a photodiagnosis (PD) followed by a fluorescence-guided resection (FGR) and completed by an intraoperative PDT. Right side: Clinical imaging assistance plays an essential role in brain tumour PDT, including estimating the initial tumour volume, 3D treatment planning (light dose according to the volume geometry), and outcome assessment. Computed 3D image-based treatment planning provided reproducible data-based tumour volumes, stereotactic accuracy for tumour volume resection, and interstitial light fibre insertion for iPDT. Optical fibres for PDT can be placed under contrast-enhanced computed tomography (CT) guidance, and the surgical approach and trajectories can be planned using preoperative diffusion magnetic resonance imaging (MRI), CT imaging, and/or single-photon emission computed tomography (SPECT) images to achieve precise tumour localization, realize a minimally invasive craniotomy, and minimize controlateral brain injury. Reproduced with permission from D. Bechet, Cancer Treat. Rev. 2014 [88].

For this reason, PDT has been proposed as a complementary approach to conventional therapies to reduce recurrence and extend survival with minimal side effects [88,90]. Technologies for brain cancer include interstitial PDT and fluorescence-guided resection (FGR). In Figure 3, the clinical possibilities of PDT can be observed. The latter reference summarizes several in vitro and in vivo studies, highlighting the PDT effect on the blood–brain barrier (BBB) and the brain-adjacent-to-tumour region. In this regard, some studies reveal a PDT-induced opening of the BBB for drug-brain delivery during post-surgical treatment of glioblastoma [92]. Furthermore, results about some PS can be found in [89], e.g., the promising results of using hematoporphyrin derivative (HpD), Talaporfin with a cavitary light application, and Foscan for fluorescence-guided resection (FGR). Additionally, 5-Aminolevulinic acid-FGR with postop cavitary HpD PDT has shown better results than control, but there is not a proper comparison to standard of care [89]. Another systematic review of the main clinical studies in the field of fluorescent diagnostics and intraoperative photodynamic therapy of primary, recurrent, and metastatic forms of malignant brain tumours can be found in [91], but it is not translated to English.

#### 2.2.8. Bladder Cancer

Bladder cancer includes several types of cancer arising from the tissues of the urinary bladder. Depending on the stage, it can be classified as non-muscle-invasive (the majority of detected cases), muscle-invasive, or metastatic. This kind of cancer was responsible for PDT resurgence in 1976, when human bladder tumour cells were transplanted into mice and destroyed for the first time using Photofrin [93]. In the latter reference, it was concluded that PDT may be recommended as a second-line or immediate therapy for patients for whom multiple transurethral resections, chemotherapy, and/or intravesical Bacillus Calmette Guerin (BCG) immunotherapy alone had failed [93]. The selection of the proper photosensitizer for bladder PDT is still debated. So far, hexylester of ALA (HAL) is the only drug that has been approved in Europe for clinical use. However, this drug has a relatively low tumour-specific uptake and suffers from photodegradation upon irradiation, which might hamper PDT efficacy. Therefore, Hypericin has been suggested to be a good alternative. In Japan, ALA has been used in Phase III trials, concluding that it might be useful for the treatment of patients suffering from carcinoma in situ when BCG therapy is not suitable. Despite this, the relatively low efficiency of ALA-PDT with blue- or red-light irradiation through water in the bladder has to be improved [94]. Despite the fact that PDT has demonstrated a high effectivity in non-muscle bladder cancer, the adverse effects limit its clinical implementation. Lee et al. [100] collected all possible adverse effects from different clinical trials, and bladder contracture, bladder fibrosis, skin photosensitivity, reduction and loss in bladder capacity, irritating lower urinary tract symptoms (frequency and urgency), and asymptomatic reflux were some of the more alarming effects seen with first-generation PSs (Photofrin and hematoporphyrin) [95]. As noted in the latter reference, 5-ALA resulted in dysuria, frequency, and urgency, and although HAL did not result in any serious effect, it lacked the efficacy of newer agents such as Radachlorin. However, very few studies conducted so far have analysed the therapeutic effects of HAL. The predominant reason for adverse effects and toxicity from PS is the non-specific accumulation of PSs in the normal urothelium. Consequently, “back-reflected” light can activate the PSs in normal urothelium, leading to tissue destruction and therefore the aforementioned side effects [95].

### 2.3. Photosensitizers

**Figure 4:**
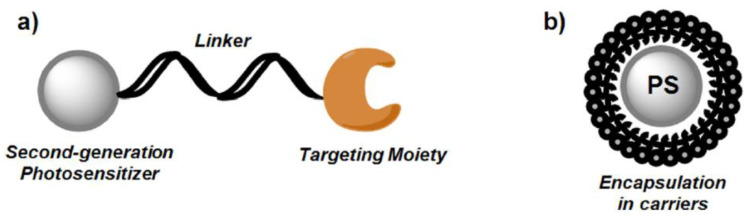
General design of 3rd generation PS: (**a**) conjugation of second-generation PS with targeting moiety; (**b**) encapsulation of the second generation into carriers (liposomes, micelles, and nanoparticles). Reproduced with permission from Ivan S. Mfouo-Tynga et al., Photodiagnosis and Photodynamic Therapy; published by Elsevier, 2021 [124].

A PS is a molecule that produces a chemical change in another molecule in a photochemical process. Photosensitizers generally work by absorbing the ultraviolet or visible region of electromagnetic radiation and transferring it, with a certain efficiency, to adjacent molecules. Because PSs are key components of PDT, this field is also one of the most important and investigated. Despite there being many specific topics, such as PS mechanisms [101,102,103,104,105] specific PSs [106,107,108,109,110,111,112,113,114,115,116], or new PSs [117,118,119,120,121,122,123] for NIR radiation, in recent years, research efforts have focused on advanced PSs (the so-called third generation). Bioconjugation and encapsulation are the main strategies behind these novel PSs (Figure 4), which could increase the specificity of phototoxic effects on targeted pathological tissue [124]. 

**Figure 5:**
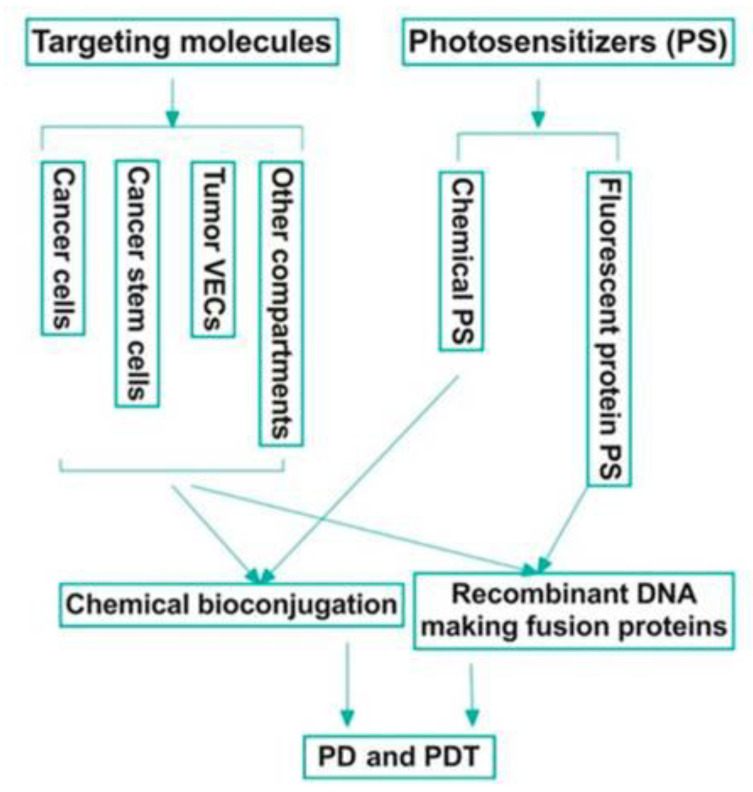
Bioconjugation strategies in photodiagnosis (PD) and therapy (PDT) of cancer. An ideal target, such as tissue factor (also known as CD142), should be commonly yet selectively expressed by multiple tumour compartments, including but not limited to cancer cells, cancer stem cells, and tumour vascular endothelial cells (VCEs), whereas it is negatively, minimally, or restrictedly expressed in normal cells. A potentially new bioconjugation strategy is the use of recombinant DNA techniques to produce fusion proteins that contain a targeting domain and a fluorescent protein PS simultaneously for targeted PD and PDT. Reproduced with permission from S. Gomez et al., Molecules; published by MDPI, 2020 [125].

As was noted in Ref. [125], the bioconjugation follows a well-established strategy, consisting of chemical conjugation of a targeting molecule to a chemical PS (with direct or indirect linkage) or a bioconjugated organic PS (with a targeting peptide or antibodies), as seen in Figure 5. The molecule can be a peptide, a full length or partial sequence of a ligand (for a membrane-bound receptor), or a full-length antibody or antibody fragments (also called photoimmunoconjugates [126,127,128]). An ideal tumour target molecule would be commonly expressed on several malignancy-related compartments in the tumour microenvironment, including, but not limited to, cancer cells, tumour vascular endothelial cells (VCEs), cancer stem cells, and myeloid-derived suppressor cells [125].

Alternatively, the use of nanotechnology in PDT has undergone exponential growth in the last decade (see Figure 1). Typically, nanospheres and nanocapsules with diameters lower than 100 nm are employed to act as drug carriers. Some advantages of nanocapsules and nanospheres were pointed out in Ref. [129]: (i) they can transport hydrophobic drugs in blood; (ii) their surface area can be modified with functional groups for additional chemical/biochemical properties; (iii) a controlled release of drug is possible; (iv) they have large distribution volumes and are generally taken up efficiently by cells; and finally, (v) a high number of different synthetic strategies are available. The first review on this topic can be found in 2007 [130]. The following year, two remarkable reviews were published. One was based on quantum dots and nanoparticles [131] (463 citations), and the other one was focused on self-lighting nanoparticles [132] (96 citations). Later, several reviews about nanotechnology were published [133,134,135,136,137,138,139,140,141,142,143,144,145], one of them also focused on photothermal therapy [146]. Apart from second-generation PS with improved selectivity and reduced toxicity, the third generation promises a total targeted therapy, in which PS can be selectively attached to specific tumour cells [147,148]. This is of paramount importance to reduce toxicity to normal cells (limiting off-target effects) and even to avoid one of the main drawbacks of many PSs used in PDT, i.e., the photosensitivity period. This targeted PS can also surpass the limited tissue penetration of current compounds [149]. In this regard, better tumour infiltration and more homogeneous distribution have been seen after the use of targeting strategies, with an improved tumour eradication [24].

Presently, the number of approved PS has grown considerably, and in Table 2, some of them are summarized. In addition, readers can check a more detailed table in the Supplemental Material Table S1 (more PS and some important characteristics such as λ_exc_, time to PDT after dose delivery, clearance, clinical/preclinical application, side effects, localization, primary mechanism of action, and manufacturer).

### 2.4. PDT Mechanisms

PDT mechanisms include ROS generation, cell death, vascular damage, and immune response, among others. In the case of ROS generation, a complete and comprehensive review can be found in [151]. Regarding cell death, the principal consequences of PDT are apoptosis, paraptosis, necrosis, and autophagy (the latter usually considered to be cytoprotective) [152]. In this context, three focused reviews about this topic were published in 2005 [153,154,155]. The first reference included a brief introduction on basic PDT principles (light sources, PS, photochemistry, and some clinical applications), and the cell death and signalling mechanisms were thoroughly studied. The second reference was focused on apoptosis mechanisms after PDT. The experimental variables that determine the cellular response and some mechanisms of the induction and production of apoptosis were discussed in detail. Finally, Ref. [155] is the most relevant in terms of the number of citations (466). It was the second of a series of three reviews, in which the mechanisms operating in PDT are discussed on a cellular level. In the same topic, a short review was published in 2009 [156]. Apart from reviewing both the cell death and tumour destruction mechanisms, an interesting discussion on signal transduction pathways was included (calcium expression levels, lipid metabolism effects, tyrosine kinase expression, transcription factors, cell adhesion molecules, and cytokines). Two years later, another review also addressed the three forms of cell death and described the critical players that control cell death related to PDT (see Table 3) [157]. In the case of paraptosis, D. Kessel has worked extensively in this death pathway and also has been discussed it in his recent reviews [158,159].

**Figure 6:**
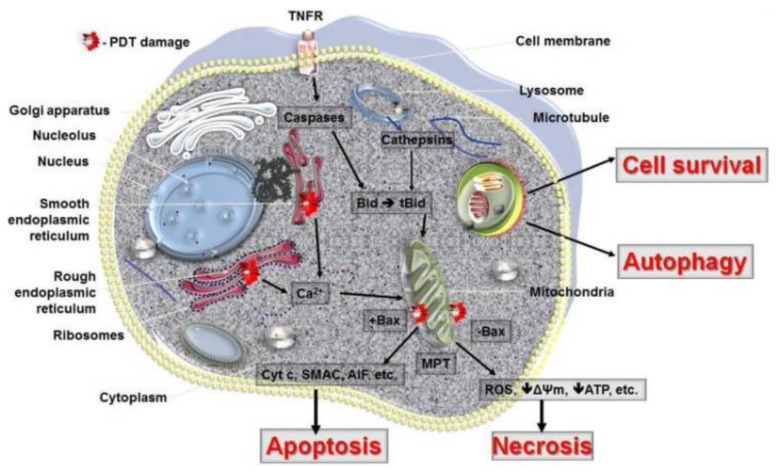
Cell death pathways in PDT. The mode of cell death observed after PDT depends to some extent on the intracellular localization of the PS and PDT-related damage to that organelle. PDT with PS localized in mitochondria will lead to loss of membrane permeability and release of pro-apoptotic mediators, while ER damage will release cellular deposits of calcium. PS that accumulates in lysosomes will release proteolytic enzymes upon illumination. Lysosomes may also fuse with autophagosomes to hydrolyse-damaged organelles and recycle them during autophagy. In the case of excess of damage, the cell will not survive despite the initiation of autophagy. Necrosis and autophagy may be dominant cell death modes after PDT when apoptosis is dysfunctional. It should be remembered that several PS may localize in more than one organelle, and the activation of cell death pathways may occur concurrently. Reproduced with permission from Pawel Mroz et al., Cancers; published by MDPI, 2011 [157].

In addition, the following years brought four similar reviews about this topic [160,161,162,163,164]. As stated in all of these reviews, despite mechanisms of cell death being identified (apoptosis, necrosis, and autophagy; see Figure 6 [157]), the response to PDT can vary due to many factors: for example, the cell type, its metabolism and genetics, the experimental model, the light dose, the type of PS, and most importantly, its intracellular localization [157]. As can be observed in Figure 6, this localization will determine which cell death pathway is initially activated [165]. The amount of damage it receives will also determine the cellular response. Other important studies have been focused on the tumour suppressor p53 protein [166], some angiogenesis inhibitors [167], and pharmacological inhibition strategies to enhance the therapeutic efficacy of PDT [168].

As commented on by [156], the diverse mutations in cancer cells caused by the expression of oncogenes may lead to an increased or decreased susceptibility to PDT, when considering the different molecular and cellular pathways. However, it is thought that many mutations that lead to resistance to radiation or chemotherapy do not necessarily lead to resistance after PDT treatment. This could be a clear advantage when conventional treatments can no longer be applied. Other reports also suggest additive benefits for PDT when combined with ionizing radiation and chemotherapy [65,169].

Furthermore, it is well known that PDT can activate the immune system through Damage-Associated Molecular Patterns (DAMPs) released by or exposed to dying cells [165,170,171,172,173,174,175,176,177], which in turn stimulate both innate and adaptive immunity (there are several reports of patients who survived much longer than expected). The potential of this fact is enormous, taking into account that common cancer therapies tend to be immunosuppressive. PDT could be an ideal cancer treatment, capable of destroying the tumour and sensitizing the immune system to metastasis [178,179]. The problem is that this effect is not always observed. This could be explained by the ability of many tumours to suppress the host immune system and to actively evade immune attacks, causing robust immune responses after PDT to remain the exception rather than the rule [180]. The latter reference concluded that the expression of the right kind of antigen in the tumour is of critical importance to promote beneficial immune system response. One solution is to overcome the immunosuppressive mechanisms that allow the tumour to grow (for example, by using immunostimulatory adjuvants, strategies that involve dendritic cells, depletion of Tregs, and epigenetic reversal agents). In any case, another important and negative consequence of PDT to have in mind is hypoxia, which often occurs in the tumour microenvironment [181,182,183,184]. Oxygen self-sufficient [185] and oxygen-boosted PDT could be the solutions. Some strategies involve oxygen carriers or generators, such as nanoparticles based on perfluorocarbon or manganese dioxide (MnO_2_) and haemoglobin-based nanostructures [186]. Another strategy could be the use of low-fluence light to avoid oxygen depletion. As noted in [177], high fluence seems to negatively impact immune reactivity towards tumour antigens in patients with basal cell carcinoma. A potent vascular shutdown preventing immune cells from reaching the tumour area is one possible explanation of such negative impact. Alternatively, an enhanced efficacy in treating patients with superficial basal cell carcinoma has been observed when it is used a fractionated illumination strategy (low fluence followed by high fluence) [187]. This suggests that great clinical benefit may arise from the design of two-step PDT programs consisting of the first immune-stimulating illumination regimen, followed by a regimen that mediates potent tumour destruction, which has proven favourable in the preclinical setting [188]. Overall, PDT still has quite a long road to travel in this respect, as many of these PDT-based program combinations must be tested and optimised in clinical settings [189,190].

In conclusion, a better understanding of the cellular mechanisms will allow predicting the tumorigenic response to treatment. This will also shed light on the myriad of possible combination treatments.

### 2.5. Dosimetry and Sources

In this section, after a brief introduction to dosimetry, the most recent reviews related to dosimetry for PDT are described. Then, a compilation of main reviews regarding light sources is given. Few dedicated reviews exist for dosimetry and light sources, whereas a larger number of reviews exist related to optical imaging. Some reviews are not explicitly mentioned here. They have been excluded mainly because the latest reviews covered contain synthesized information of—or reference to—previous reviews on the topic.

Dosimetry involves a series of measurements of the PDT dose for pre-planning, real-time monitoring, and adaptation to the treatment through outcome assessment. It can be subdivided into different categories: explicit, implicit, direct, and biophysical tissue response monitoring [191,192,193]. In general, the PDT dose depends directly on the light fluence, photosensitizer concentration, and oxygen concentration. Several other factors have been identified as potential modifiers to the PDT dose, including oxygen distribution and photosensitizer distribution, singlet oxygen generation properties, photobleaching rate, blood flow, system immune response, optical tissue properties, light delivery, fractionation, and drug-light interval, among others [194,195]. Most of these factors—or parameters—have a dynamic response and may exhibit different distributions microscopically (e.g., oxygenation may vary orders of magnitude every few tenths of a micrometre [194]), and are subjected to patient-to-patient variations. All these factors influencing PDT dose call attention to the importance and high degree of complexity for implementation of dosimetry in the clinic.

**Figure 7:**
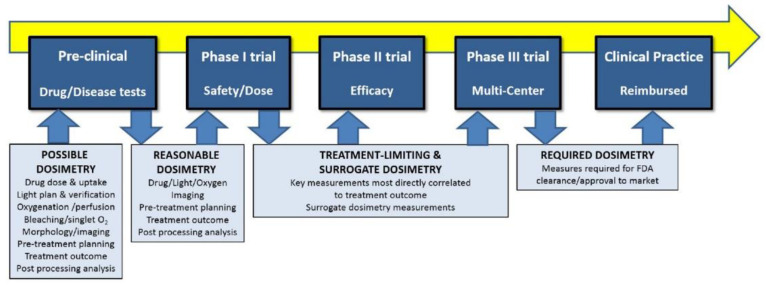
The shift from preclinical evaluation, through Phase I, II, and III clinical trials, and finally to accepted clinical practice is illustrated, with the advocated shifts in dosimetry goals throughout the pathway. While a comprehensive dosimetry approach might be used in preclinical work to mechanistically inform practice, the transition to Phase I trial requires that reasonably efficient methods of dosimetry be implemented, and in the future, the goal should be to shift towards treatment-limiting dosimetry and correlated surrogates. Eventually, these might become the required dosimetry in approved/cleared treatments, going beyond basic prescriptions of light and drug doses. The image and caption are reproduced from their original created by Pogue, B.W. et al., Phys. Med. Biol. 2016, 61, R57–R89 (doi:10.1088/0031-9155/61/7/R57) [194] under Creative Commons Attribution 3.0.

Presently, it is well-known that dosimetry is of paramount importance and has a major impact on PDT efficacy [191,194,195]. Real-time dosimetry providing optimized and adapted treatments has shown great potential in certain cases (e.g., prostate [196]); however, there are still several challenges. Most of these challenges and pathways for future research have been summarized in recent reviews. In 2016, a comprehensive review revisiting the dosimetry for PDT was published [194]. It provides an excellent overview of the main challenges in dosimetry with a strong focus on clinical transfer. A succinct and detailed description and implications of the main parameters involved in dosimetry and the methods for their determination were provided. Specifically, some of these challenges are (i) uncertainty of photosensitizer concentration, location, and effect in vivo, (ii) estimates of light dose difficulties involving the PS, light, and oxygen in bulky tissues (e.g., the need to predict dose parameters between measurement probes), (iii) complete models of PDT dose (some measurements are still needed for reliable modelling), and (iv) biological modifiers of PDT dose. Since it is widely recognized that performing full dosimetry is a highly challenging task [194], especially for clinical transfer, the authors discussed and presented examples of a more realistic implementation of dosimetry in clinical settings. Such implementation may be based on reductionist and surrogate approaches, with possible full dosimetry implementation in preclinical evaluation (Figure 7) [194]. Reductionist dosimetry is based on the careful identification of key parameters determining the PDT dose (may not always be possible), whereas surrogate dosimetry would use clinical tools available.

Specifically, in 2016, Li et al. reviewed the status and outlook focusing only on singlet oxygen generation and detection techniques [197]. A more recent review (2020), including a short dosimetry section, can be found in [193]. To complement Pogue et al.’s review [194], additional evidence regarding the importance of dosimetry has been gathered, including practical examples of explicit and implicit dosimetry, e.g., implicit dosimetry performed in intraoperative pleural PDT. Simplified rate equations for the dose model were provided, which are also linked to a review useful for dosimetry on the modelling of in vivo photochemical properties of PDT reactive oxygen species and describing the techniques to measure them [198].

Dosimetry also involves the use of optical imaging techniques, including structural, functional, and molecular imaging. Optical imaging is not only essential for PDT dose assessment but also for pursuing a better understanding of key factors affecting PDT. Many of the effects influencing PDT often occur at localized regions and at the micrometre level, for which optical imaging techniques have become essential. In this context, optical imaging techniques have shown great potential for tissue diagnosis, pre-treatment planning, imaging-guided therapy (e.g., fluorescence guiding), monitoring, and outcome assessment [199,200,201,202]. As compared to conventional techniques (e.g., magnetic resonance imaging), optical imaging techniques provide real-time feedback and more accurate and reliable detection of tumour volumes of less than 1 cm [202]. A complete and thorough review was published in 2010 [200]. It included the photochemical and photophysical fundamentals, a description of different imaging modalities, clinical/preclinical studies related to the detection of cancer or precancerous growth, the guidance of surgical resection, and monitoring of treatment response. In 2015, Mallidi et al. published a focused review on optical imaging and combined treatments for PDT, highlighting the role of optical imaging in the structural, functional (e.g., blood oxygen saturation has been used as an indicator of PDT efficacy), and molecular information on PDT action [199]. Although it is not a review *per se*, it is also worth mentioning a dedicated book on imaging in PDT with full information about the microscopic effects of many factors influencing PDT dose (e.g., photosensitizer activity, localization, structural and functional changes in tumour vasculature, clinical imaging, etc.) [202]. More recently (2020), De Silva et al. included a section compiling results about optical imaging techniques and imaging-guided therapies for PDT, and Hester et al. published a review focused on photoacoustic and ultrasound imaging for PDT [203]. Cutting-edge nonlinear multiphoton microscopy techniques have also shown great potential to provide detailed biological imaging of the tumour microenvironment (and the possibility for label-free imaging), allowing a better understanding of cellular biophysical and biological processes. Some of these nonlinear techniques have proven their validity for PDT [204]. A recent review (2020) on these techniques (not linked to PDT) has been published by Parodi et al. [205].

Regarding light sources, selected reviews include conventional light sources for interstitial and superficial applications [193,206,207,208,209,210]. Recently, deep PDT has gained interest, and several published reviews can be found. In 2015, a review of nanocomposite-based strategies for deep PDT was published [211]. Additionally, the three reviews of 2016 are remarkable in terms of depth of contents, including particular depth concerning PDT light sources [210,212,213]. They are focused on novel multifunctional nanoparticles, which can be classified into three major groups: near-infrared (NIR) light-excited nanomaterials [214,215,216], X-ray/Cherenkov excited scintillating/afterglow nanoparticles [217,218,219,220,221,222], and self-illuminated nanoconjugates [212,223]. Recently, implants have experienced rapid development, and several proofs of concepts with diverse modes of activation and light sources have been reported [224,225,226,227,228,229]. To our knowledge, there is no dedicated review of implants for PDT. A section devoted to the description and critical review of selected implants can be found in a recent review of light technology for PDT [230].

## 3. Summary and Conclusions

This contribution has compiled the latest PDT review articles from the last two decades. The recent exponential increase in publications on PDT is due to the advance of material science, with state-of-the-art PSs capable of detecting and affecting only diseased cells. The number of clinical trials has increased in recent years, indicating that the benefits of PDT remain attractive and promising and that the field is very active. In some cases, PDT exhibits better outcomes for smaller lesions than bigger or deeper lesions, as compared to surgery. Regarding the comparison with chemotherapy or radiotherapy, PDT has proved to be an effective and safe alternative with lower morbidity. 

Advancements in formulations and nanotechnology have enabled enhanced and new developments of PSs. Apart from second-generation PS with improved selectivity and reduced toxicity, the third generation pursues a total targeted therapy, in which PS can be selectively attached to specific tumour cells. Reduced toxicity, better tumour infiltration, and more homogenous distribution have been demonstrated, improving tumour eradication. 

It is also worth mentioning the deep knowledge about different PDT mechanisms that have been acquired in these years, understanding more and more key processes, such as the activation of the immune system. The latter and related processes are of vital importance for predict ingthe tumorigenic response to treatment and develop new PS capable of localizing in specific regions of the cell, or vaccines against cancer.

Considering the key points and main conclusions drawn from the articles mentioned in this paper, the future incorporation of PDT into routine cancer treatments in the clinical setting is forecasted, either as part of a multimodality approach or as a single treatment against early cancer or palliative treatment. It should be noted that the new generation of PSs, both bioconjugation and encapsulation, as well as better source-based planning and dosimetry, can make PDT a competitive rival to conventional therapies. To conclude, the latest clinical trials have shown the enormous potential of PDT, while preclinical trials indicate that there is still much room for improvement on many fronts.

## Figures and Tables

**Table 1:** Reviews of the last decade for the most studied types of cancers in the PDT field.

Type of Cancer	Year	Ref.	Content
Gastrointestinal (liver and bile ducts, pancreas, small intestine, colon, and rectum)	2011	[31]	State-of-the-art on cholangiocarcinoma.
	2012	[32]	Unresectable cholangiocarcinoma.
	2013	[33]	PDT in gastroenterology.
	2015	[34,35]	Cholangiocarcinoma; colorectal clinical trials.
	2016	[36,37]	Pancreas PDT; colorectal preclinical research.
	2017	[38,39]	Cholangiocarcinoma; colon cancer and stem cells.
	2019	[40]	Colon (immunotherapy and PDT).
	2020	[41]	Liver malignancies.
	2021	[42,43]	Colorectal (PDT and cannabidiol); pancreas (PS nanoparticles).
Skin	2010	[44,45]	Skin and other types of cancer reviews.
	2015	[46,47]	MTHPC for non-melanoma skin cancers; combined strategy.
	2016	[48,49,50]	Non-melanoma skin cancer.
	2018	[51,52]	Immune consequences induced by PDT; non-melanoma.
	2019	[53]	Dermato-oncology.
	2020	[54,55]	Methyl-5-aminolevulinate for basal cell carcinoma; mechanisms, challenges, and promising developments
Lung	2011	[56,57,58]	General review; early-stage; targets and mechanisms.
	2012	[59,60]	Non-small-cell lung cancer, Kato’s 30-year experience.
	2014	[61]	Lung cancer and malignant pleural mesothelioma.
	2016	[62]	Non-small-cell lung cancer, review and future directions.
	2018	[63]	Nanoparticle PS drug delivery uptake systems.
	2021	[64,65]	Update; chemotherapy and PDT in lung cancer.
Prostate	2010	[66]	PDT for focal ablation of the prostate.
	2011	[67]	Perspective.
	2015	[68]	WST-09 and WST-11 mediated vascular-targeted PDT.
	2017	[69]	Photosensitizers in prostate cancer therapy.
	2019	[70,71,72]	Low-risk prostate cancer, review and meta-analysis.
	2021	[73,74]	Narrative review; comparison with minimally invasive techniques.
Head and neck	2011	[75]	Mucosal dysplasia and microinvasive carcinoma.
	2012	[76]	5-Aminolevulinic acid-mediated PDT.
	2013	[77]	MTHPC mediated squamous cell carcinoma.
	2015	[78]	Oral premalignant lesions.
	2016	[79]	Oral cancer and premalignant lesions.
	2014	[80]	Detailed review about PS.
	2018	[81]	Neoplasms of the head and neck.
	2019	[82]	Indications, outcomes, and prospects.
Breast	2016	[83]	The plant-derived agent-induced cell death mechanisms.
	2017	[84]	PDT: inception to application in breast cancer.
	2019	[15,85]	Multidrug-resistant; intradermal metastatic breast cancer.
	2020	[86]	Organic nanoparticle-based active targeting for PDT.
	2021	[87]	Perspective.
Brain	2014	[88]	General review (sources, dosimetry, PS, etc).
	2015	[89]	Perspective.
	2016	[90]	Current status and prospects of PDT in Japan.
	2018	[91]	A systematic review of clinical trials.
	2020	[92]	Complete review of PDT and novel OCT strategies.
Bladder	2011	[93]	Overview of preclinical and clinical experiences.
	2017	[94]	5-Aminolevulinic acid-mediated PDT.
	2018	[95]	Past challenges and current innovations.

**Table 2:** Photosensitizers approved or under clinical trials [150]. A more detailed table can be found in the Supplemental Material, Table S1.

Name	λ_exc_ (nm)	Manufacturer	Application
FIRST-GENERATION PHOTOSENSITIZERS			
Porfimer sodium	630	Axcan Pharma	PDT of esophageal cancer, lung adenocarcinoma, and endobronchial cancer
SECOND-GENERATION PHOTOSENSITIZERS/Prodrugs			
5-aminolaevulinic acid	635	DUSA Stabiopharma	PDT of mild to moderate actinic keratosis, with fluorescence-guided resection of glioma
Methyl-aminolevulinic acid	579–670	Galderma	PDT of non-hyperkeratotic actinic keratosis and basal cell carcinoma
Temoporfin	652	Biolitec	PDT of advanced head and neck cancer
Talaporfin	664	Meiji SeikaNovartis	PDT of early centrally located lung cancer
Verteporfin	690	Novartis	PDT of age-related macular degeneration
Redaporfin	749	Luzitin	PDT of biliary tract cancer
PHOTOSENSITIZERS UNDER CLINICAL INVESTIGATIONS			
Fotolon	665	Apocare Pharma	PDT of nasopharyngeal sarcoma
Hexylaminolevulinate	635	Photocure	PDT of HPV-induced cervical precancerous lesions and non-muscle invasive bladder cancer
Radachlorin	662	Rada-pharma	PDT of skin cancer
Photochlor (HTTP)	664	Rosewell Park	PDT of head and neck cancer
Padeliporfin	762	Negma-Lerads	PDT of prostate cancer
Motexafin lutetium	732	Pharmacyclics	PDT of coronary artery disease
Rostaprofin	664	Miravant	PDT of age-related macular degeneration
Talaporfin	664	Meiji Seika	PDT of colorectal neoplasms, liver metastasis
Fimaporfin	435	PCI Biotech	PCI of cutaneous or sub-cutaneous malignancies, cholangiocarcinoma, and PCI of vaccine antigens

**Table 3:** Major cell death mechanism activated by PDT. Reproduced with permission from Pawel Mroz et al., Cancers; published by MDPI, 2011 [157].

Anti-Tumour PDT Mechanisms		
	Organelles	Processes
Direct cell damage	Mitochondria:	Apoptosis
	- Cytochrome c release - Bcl-2 damage	
	Cytoplasm: - NFkB damage	
	Endoplasmatic reticulum - Beclin 1 - mTOR activation	Autophagy
	Cell membrane disintegration	Necrosis
Vascular shutdown	Local depletion of oxygen and nutrients	Apoptosis Necrosis Autophagy
Activation of immune response	Cytotoxic T cells	Granzyme mediated apoptosis

## Supplementary Materials

The following are available online at https://www.mdpi.com/article/10.3390/cancers13174447/s1, Table S1: Main characteristics of some common photosensitizers.

## References

[1] Ferlay J., Ervik M., Lam F., Colombet M., Mery L., Piñeros M. (2018). Global Cancer Observatory: Cancer Today.

[2] Hamblin M.R. (2020). Photodynamic Therapy for Cancer: What’s Past Is Prologue. Photochem. Photobiol..

[3] Ackroyd R., Kelty C., Brown N., Reed M. (2001). The History of Photodetection and Photodynamic Therapy. Photochem. Photobiol..

[4] Prime J. (1900). Les Accidentes Toxiques Par L’eosinate de Sodium.

[5] Tappeiner H., Jesionek A. (1903). Therapeutische Versuche Mit Fluoreszierenden Stoffen. Muench. Med. Wochenschr..

[6] Tappeiner H., Jodlbauer A. (1907). Die Sensibilisierende Wirkung Fluorieszierender Substanzer. Gesammte Untersuchungen Uber Die Photodynamische Erscheinung.

[7] Diamond I., Mcdonagh A.F., Wilson C.B., Granelli S.G., Nielsen S., Jaenicke R. (1972). Photodynamic Therapy of Malignant Tumours. Lancet.

[8] Dougherty T.J., Grindey G.B., Fiel R., Weishaupt K.R., Boyle D.G. (1975). Photoradiation Therapy. II. Cure of Animal Tumors with Hematoporphyrin and Light. J. Natl. Cancer Inst..

[9] Kelly J.F., Snell M.E. (1976). Hematoporphyrin Derivative: A Possible Aid in the Diagnosis and Therapy of Carcinoma of the Bladder. J. Urol..

[10] Dougherty T.J., Kaufman J.E., Goldfarb A., Weishaupt K.R., Boyle D., Mittleman A. (1978). Photoradiation Therapy for the Treatment of Malignant Tumors. Cancer Res..

[11] U.S. National Library of Medicine. https://clinicaltrials.gov/.

[12] Moan J., Berg K. (1992). Photochemotherapy of Cancer: Experimental Research. Photochem. Photobiol..

[13] Moan J., Peng Q. (2003). An Outline of the Hundred-Year History of PDT. Anticancer Res..

[14] Barr H., Tralau C.J., Boulos P.B., MacRobert A.J., Tilly R., Bown S.G. (1987). The Contrasting Mechanisms of Colonic Collagen Damage between Photodynamic Therapy and Thermal Injury. Photochem. Photobiol..

[15] Aniogo E.C., Plackal Adimuriyil George B., Abrahamse H. (2019). The Role of Photodynamic Therapy on Multidrug Resistant Breast Cancer. Cancer Cell Int..

[16] Spring B.Q., Rizvi I., Xu N., Hasan T. (2015). The Role of Photodynamic Therapy in Overcoming Cancer Drug Resistance. Photochem. Photobiol. Sci..

[17] Kleinovink J.W., Van Driel P.B., Snoeks T.J., Prokopi N., Fransen M.F., Cruz L.J., Mezzanotte L., Chan A., Löwik C.W., Ossendorp F. (2016). Combination of Photodynamic Therapy and Specific Immunotherapy Efficiently Eradicates Established Tumors. Clin. Cancer Res..

[18] MacDonald I.J., Dougherty T.J. (2001). Basic Principles of Photodynamic Therapy. J. Porphyr. Phthalocyanines.

[19] Wilson B.C. (2002). Photodynamic Therapy for Cancer: Principles. Can. J. Gastroenterol..

[20] Dolmans D.E.J.G.J., Fukumura D., Jain R.K. (2003). Photodynamic Therapy for Cancer. Nat. Rev. Cancer.

[21] Brown S.B., Brown E.A., Walker I. (2004). The Present and Future Role of Photodynamic Therapy in Cancer Treatment. Lancet Oncol..

[22] Wilson B.C., Patterson M.S. (2008). The Physics, Biophysics and Technology of Photodynamic Therapy. Phys. Med. Biol..

[23] Agostinis P., Berg K., Cengel K.A., Foster T.H., Girotti A.W., Gollnick S.O., Hahn S.M., Hamblin M.R., Juzeniene A., Kessel D. (2011). Photodynamic Therapy of Cancer: An Update. CA Cancer J. Clin..

[24] van Straten D., Mashayekhi V., de Bruijn H.S., Oliveira S., Robinson D.J. (2017). Oncologic Photodynamic Therapy: Basic Principles, Current Clinical Status and Future Directions. Cancers.

[25] Dos Santos A.F., De Almeida D.R.Q., Terra L.F., Baptista M.S., Labriola L. (2019). Photodynamic Therapy in Cancer Treatment–an Update Review. J. Cancer Metastasis Treat..

[26] Yang M., Yang T., Mao C. (2019). Enhancement of Photodynamic Cancer Therapy by Physical and Chemical Factors. Angew. Chem. Int. Ed..

[27] RL Y., DW B., GS R., SJ I., ST C. (2019). Photodynamic Therapy for Solid Tumors: A Review of the Literature. Photodermatol. Photoimmunol. Photomed..

[28] Kessel D. (2019). Photodynamic Therapy: A Brief History. J. Clin. Med..

[29] Li X., Lovell J.F., Yoon J., Chen X. (2020). Clinical Development and Potential of Photothermal and Photodynamic Therapies for Cancer. Nat. Rev. Clin. Oncol..

[30] Gunaydin G., Gedik M.E., Ayan S. (2021). Photodynamic Therapy for the Treatment and Diagnosis of Cancer–A Review of the Current Clinical Status. Front. Chem..

[31] Ortner M.-A. (2011). Photodynamic Therapy for Cholangiocarcinoma. Lasers Surg. Med..

[32] Tomizawa Y., Tian J. (2012). Photodynamic Therapy for Unresectable Cholangiocarcinoma. Dig. Dis. Sci..

[33] Shishkova N., Kuznetsova O., Berezov T. (2013). Photodynamic Therapy in Gastroenterology. J. Gastrointest. Cancer.

[34] Patel J., Rizk N., Kahaleh M. (2015). Role of Photodynamic Therapy and Intraductal Radiofrequency Ablation in Cholangiocarcinoma. Best Pract. Res. Clin. Gastroenterol..

[35] Kawczyk-Krupka A., Bugaj A.M., Latos W., Zaremba K., Wawrzyniec K., Sieroń A. (2015). Photodynamic Therapy in Colorectal Cancer Treatment: The State of the Art in Clinical Trials.

[36] Bown S.G. (2016). Photodynamic Therapy for Cancer of the Pancreas-The Story so Far. Photonics Lasers Med..

[37] Kawczyk-Krupka A., Bugaj A.M., Latos W., Zaremba K., Wawrzyniec K., Kucharzewski M., Sieroń A. (2016). Photodynamic Therapy in Colorectal Cancer Treatment-The State of the Art in Preclinical Research. Photodiagnosis Photodyn. Ther..

[38] Moole H., Tathireddy H., Dharmapuri S., Moole V., Boddireddy R., Yedama P., Dharmapuri S., Uppu A., Bondalapati N., Duvvuri A. (2017). Success of Photodynamic Therapy in Palliating Patients with Nonresectable Cholangiocarcinoma: A Systematic Review and Meta-Analysis. World J. Gastroenterol..

[39] Hodgkinson N., Kruger C.A., Abrahamse H. (2017). Targeted Photodynamic Therapy as Potential Treatment Modality for the Eradication of Colon Cancer and Colon Cancer Stem Cells. Tumor Biol..

[40] Kaleta-Richter M., Kawczyk-Krupka A., Aebisher D., Bartusik-Aebisher D., Czuba Z., Cieślar G. (2019). The Capability and Potential of New Forms of Personalized Colon Cancer Treatment: Immunotherapy and Photodynamic Therapy. Photodiagnosis Photodyn. Ther..

[41] Zou H., Wang F., Zhou J.-J., Liu X., He Q., Wang C., Zheng Y.-W., Wen Y., Xiong L. (2020). Application of Photodynamic Therapy for Liver Malignancies. J. Gastrointest. Oncol..

[42] Nkune N.W., Kruger C.A., Abrahamse H. (2021). Possible Enhancement of Photodynamic Therapy (PDT) Colorectal Cancer Treatment When Combined with Cannabidiol. Anticancer. Agents Med. Chem..

[43] Yang H., Liu R., Xu Y., Qian L., Dai Z. (2021). Photosensitizer Nanoparticles Boost Photodynamic Therapy for Pancreatic Cancer Treatment. Nano-Micro Lett..

[44] Fayter D., Corbett M., Heirs M., Fox D., Eastwood A. (2010). A Systematic Review of Photodynamic Therapy in the Treatment of Precancerous Skin Conditions, Barrett’s Oesophagus and Cancers of the Biliary Tract, Brain, Head and Neck, Lung, Oesophagus and Skin. Health Technol. Assess..

[45] Zhao B., He Y.-Y. (2010). Recent Advances in the Prevention and Treatment of Skin Cancer Using Photodynamic Therapy. Expert Rev. Anticancer Ther..

[46] Horlings R.K., Terra J.B., Witjes M.J.H. (2015). MTHPC Mediated, Systemic Photodynamic Therapy (PDT) for Nonmelanoma Skin Cancers: Case and Literature Review. Lasers Surg. Med..

[47] Lucena S.R., Salazar N., Gracia-Cazaña T., Zamarrón A., González S., Juarranz Á., Gilaberte Y. (2015). Combined Treatments with Photodynamic Therapy for Non-Melanoma Skin Cancer. Int. J. Mol. Sci..

[48] Griffin L.L., Lear J.T. (2016). Photodynamic Therapy and Non-Melanoma Skin Cancer. Cancers.

[49] Cohen D.K., Lee P.K. (2016). Photodynamic Therapy for Non-Melanoma Skin Cancers. Cancers.

[50] Erkiert-Polguj A., Halbina A., Polak-Pacholczyk I., Rotsztejn H. (2016). Light-Emitting Diodes in Photodynamic Therapy in Non-Melanoma Skin Cancers–Own Observations and Literature Review. J. Cosmet. Laser Ther..

[51] Yu X., Zheng H., Chan M.T.V., Wu W.K.K. (2018). Immune Consequences Induced by Photodynamic Therapy in Non-Melanoma Skin Cancers: A Review. Environ. Sci. Pollut. Res..

[52] Morton C.A. (2018). A Synthesis of the World’s Guidelines on Photodynamic Therapy for Non-Melanoma Skin Cancer. Ital. J. Dermatol. Venereol..

[53] Tampa M., Sarbu M.I., Matei C., Mitran C.I., Mitran M.I., Caruntu C., Constantin C., Neagu M., Georgescu S.R. (2019). Photodynamic Therapy: A Hot Topic in Dermato-Oncology (Review). Oncol. Lett..

[54] Wang B.C., Fu C., Qin L., Zeng X.Y., Liu Q. (2020). Photodynamic Therapy with Methyl-5-Aminolevulinate for Basal Cell Carcinoma: A Systematic Review and Meta-Analysis. Photodiagnosis Photodyn. Ther..

[55] Allegra A., Pioggia G., Tonacci A., Musolino C., Gangemi S. (2020). Oxidative Stress and Photodynamic Therapy of Skin Cancers: Mechanisms, Challenges and Promising Developments. Antioxidants.

[56] Allison R., Moghissi K., Downie G., Dixon K. (2011). Photodynamic Therapy (PDT) for Lung Cancer. Photodiagnosis Photodyn. Ther..

[57] Ikeda N., Usuda J., Kato H., Ishizumi T., Ichinose S., Otani K., Honda H., Furukawa K., Okunaka T., Tsutsui H. (2011). New Aspects of Photodynamic Therapy for Central Type Early Stage Lung Cancer. Lasers Surg. Med..

[58] Chiaviello A., Postiglione I., Palumbo G. (2011). Targets and Mechanisms of Photodynamic Therapy in Lung Cancer Cells: A Brief Overview. Cancers.

[59] Simone C.B., Friedberg J.S., Glatstein E., Stevenson J.P., Sterman D.H., Hahn S.M., Cengel K.A. (2012). Photodynamic Therapy for the Treatment of Non-Small Cell Lung Cancer. J. Thorac. Dis..

[60] Kato H. (2012). Our Experience with Photodynamic Diagnosis and Photodynamic Therapy for Lung Cancer. JNCCN J. Natl. Compr. Cancer Netw..

[61] Simone C.B., Cengel K.A. (2014). Photodynamic Therapy for Lung Cancer and Malignant Pleural Mesothelioma. Semin. Oncol..

[62] Shafirstein G., Battoo A., Harris K., Baumann H., Gollnick S.O., Lindenmann J., Nwogu C.E. (2016). Photodynamic Therapy of Non-Small Cell Lung Cancer Narrative Review and Future Directions. Ann. Am. Thorac. Soc..

[63] Mokwena M.G., Kruger C.A., Ivan M.T., Heidi A. (2018). A Review of Nanoparticle Photosensitizer Drug Delivery Uptake Systems for Photodynamic Treatment of Lung Cancer. Photodiagnosis Photodyn. Ther..

[64] Wang K., Yu B., Pathak J.L. (2021). An Update in Clinical Utilization of Photodynamic Therapy for Lung Cancer. J. Cancer.

[65] El-Hussein A., Manoto S.L., Ombinda-Lemboumba S., Alrowaili Z.A., Mthunzi-Kufa P. (2021). A Review of Chemotherapy and Photodynamic Therapy for Lung Cancer Treatment. Anti-cancer Agents Med. Chem..

[66] Arumainayagam N., Moore C.M., Ahmed H.U., Emberton M. (2010). Photodynamic Therapy for Focal Ablation of the Prostate. World J. Urol..

[67] Moore C.M., Emberton M., Bown S.G. (2011). Photodynamic Therapy for Prostate Cancer-an Emerging Approach for Organ-Confined Disease. Lasers Surg. Med..

[68] Kawczyk-Krupka A., Wawrzyniec K., Musiol S.K., Potempa M., Bugaj A.M., Sieroń A. (2015). Treatment of Localized Prostate Cancer Using WST-09 and WST-11 Mediated Vascular Targeted Photodynamic Therapy-A Review. Photodiagnosis Photodyn. Ther..

[69] Gheewala T., Skwor T., Munirathinam G. (2017). Photosensitizers in Prostate Cancer Therapy. Oncotarget.

[70] Kleinclauss F., Frontczak A., Balssa L., Lebdai S., Azzouzi R. (2019). Vascular Targeted Photodynamic Therapy in Low-Risk Prostate Cancer. A Literature Review. Prog. Urol..

[71] Ferroni C., Del Rio A., Martini C., Manoni E., Varchi G. (2019). Light-Induced Therapies for Prostate Cancer Treatment. Front. Chem..

[72] Wang L., Yang H., Li B. (2019). Photodynamic Therapy for Prostate Cancer: A Systematic Review and Meta-Analysis. Prostate Int..

[73] Osuchowski M., Bartusik-Aebisher D., Osuchowski F., Aebisher D. (2021). Photodynamic Therapy for Prostate Cancer–A Narrative Review. Photodiagnosis Photodyn. Ther..

[74] Guo R.-Q., Guo X.-X., Li Y.-M., Bie Z.-X., Li B., Li X.-G. (2021). Cryoablation, High-Intensity Focused Ultrasound, Irreversible Electroporation, and Vascular-Targeted Photodynamic Therapy for Prostate Cancer: A Systemic Review and Meta-Analysis. Int. J. Clin. Oncol..

[75] Quon H., Grossman C.E., Finlay J.C., Zhu T.C., Clemmens C.S., Malloy K.M., Busch T.M. (2011). Photodynamic Therapy in the Management of Pre-Malignant Head and Neck Mucosal Dysplasia and Microinvasive Carcinoma. Photodiagnosis Photodyn. Ther..

[76] Chen H.-M., Yu C.-H., Lin H.-P., Cheng S.-J., Chiang C.-P. (2012). 5-Aminolevulinic Acid-Mediated Photodynamic Therapy for Oral Cancers and Precancers. J. Dent. Sci..

[77] De Visscher S.A.H.J., Dijkstra P.U., Tan I.B., Roodenburg J.L.N., Witjes M.J.H. (2013). MTHPC Mediated Photodynamic Therapy (PDT) of Squamous Cell Carcinoma in the Head and Neck: A Systematic Review. Oral Oncol..

[78] Vohra F., Al-Kheraif A.A., Qadri T., Hassan M.I.A., Ahmed A., Warnakulasuriya S., Javed F. (2015). Efficacy of Photodynamic Therapy in the Management of Oral Premalignant Lesions. A Systematic Review. Photodiagnosis Photodyn. Ther..

[79] Saini R., Lee N.V., Liu K.Y.P., Poh C.F. (2016). Prospects in the Application of Photodynamic Therapy in Oral Cancer and Premalignant Lesions. Cancers.

[80] Nelke K.H., Pawlak W., Leszczyszyn J., Gerber H. (2014). Photodynamic Therapy in Head and Neck Cancer. Postepy Hig. Med. Dosw..

[81] Civantos F.J., Karakullukcu B., Biel M., Silver C.E., Rinaldo A., Saba N.F., Takes R.P., Vander Poorten V., Ferlito A. (2018). A Review of Photodynamic Therapy for Neoplasms of the Head and Neck. Adv. Ther..

[82] Meulemans J., Delaere P., Vander Poorten V. (2019). Photodynamic Therapy in Head and Neck Cancer: Indications, Outcomes, and Future Prospects. Curr. Opin. Otolaryngol. Head Neck Surg..

[83] Adimuriyil George B.P., Abrahamse H. (2016). A Review on Novel Breast Cancer Therapies: Photodynamic Therapy and Plant Derived Agent Induced Cell Death Mechanisms. Anti-Cancer Agents Med. Chem..

[84] Banerjee S.M., MacRobert A.J., Mosse C.A., Periera B., Bown S.G., Keshtgar M.R.S. (2017). Photodynamic Therapy: Inception to Application in Breast Cancer. Breast.

[85] Rakhimzhanova R.I., Shanazarov N.A., Turzhanova D.E. (2019). Photodynamic Therapy of Intradermal Metastatic Breast Cancer (Literature Review). Biomed. Photonics.

[86] Montaseri H., Kruger C.A., Abrahamse H. (2020). Review: Organic Nanoparticle Based Active Targeting for Photodynamic Therapy Treatment of Breast Cancer Cells. Oncotarget.

[87] Ostańska E., Aebisher D., Bartusik-Aebisher D. (2021). The Potential of Photodynamic Therapy in Current Breast Cancer Treatment Methodologies. Biomed. Pharmacother..

[88] Bechet D., Mordon S.R., Guillemin F., Barberi-Heyob M.A. (2014). Photodynamic Therapy of Malignant Brain Tumours: A Complementary Approach to Conventional Therapies. Cancer Treat. Rev..

[89] Quirk B.J., Brandal G., Donlon S., Vera J.C., Mang T.S., Foy A.B., Lew S.M., Girotti A.W., Jogal S., LaViolette P.S. (2015). Photodynamic Therapy (PDT) for Malignant Brain Tumors—Where Do We Stand?. Photodiagnosis Photodyn. Ther..

[90] Akimoto J. (2016). Photodynamic Therapy for Malignant Brain Tumors. Neurol. Med. Chir..

[91] Tzerkovsky D.A., Maslakov E.A., Bagrintsev D.A., Semak I.A., Protopovich Y.L., Chizh A.G., Tatur A.A., Fomenkov I.S., Stupak D.S. (2018). The Role of Photodynamic Therapy in the Treatment of Primary, Recurrent and Metastatic Malignant Brain Tumors. Biomed. Photonics.

[92] Abdurashitov A., Tuchin V., Semyachkina-Glushkovskaya O. (2020). Photodynamic Therapy of Brain Tumors and Novel Optical Coherence Tomography Strategies for in Vivo Monitoring of Cerebral Fluid Dynamics. J. Innov. Opt. Health Sci..

[93] Yavari N., Andersson-Engels S., Segersten U., Malmstrom P.-U. (2011). An Overview on Preclinical and Clinical Experiences with Photodynamic Therapy for Bladder Cancer. Can. J. Urol..

[94] Inoue K. (2017). 5-Aminolevulinic Acid-Mediated Photodynamic Therapy for Bladder Cancer. Int. J. Urol..

[95] Railkar R., Agarwal P.K. (2018). Photodynamic Therapy in the Treatment of Bladder Cancer: Past Challenges and Current Innovations. Eur. Urol. Focus.

[96] Wen X., Li Y., Hamblin M.R. (2017). Photodynamic Therapy in Dermatology beyond Non-Melanoma Cancer: An Update. Photodiagnosis Photodyn. Ther..

[97] National Cancer Institute, Head and Neck Cancers. https://www.cancer.gov/types/head-and-neck/head-neck-fact-sheet.

[98] Lamberti M.J., Rumie Vittar N.B., Rivarola V.A. (2014). Breast Cancer as Photodynamic Therapy Target: Enhanced Therapeutic Efficiency by Overview of Tumor Complexity. World J. Clin. Oncol..

[99] Banerjee S.M., El-Sheikh S., Malhotra A., Mosse C.A., Parker S., Williams N.R., MacRobert A.J., Hamoudi R., Bown S.G., Keshtgar M.R.S. (2020). Photodynamic Therapy in Primary Breast Cancer. J. Clin. Med..

[100] Lee J.Y., Diaz R.R., Cho K.S., Lim M.S., Chung J.S., Kim W.T., Ham W.S., Choi Y.D. (2013). Efficacy and Safety of Photodynamic Therapy for Recurrent, High Grade Nonmuscle Invasive Bladder Cancer Refractory or Intolerant to Bacille Calmette-Guérin Immunotherapy. J. Urol..

[101] Castano A.P., Demidova T.N., Hamblin M.R. (2004). Mechanisms in Photodynamic Therapy: Part One–Photosensitizers, Photochemistry and Cellular Localization. Photodiagnosis Photodyn. Ther..

[102] Castano A.P., Demidova T.N., Hamblin M.R. (2005). Mechanisms in Photodynamic Therapy: Part Three—Photosensitizer Pharmacokinetics, Biodistribution, Tumor Localization and Modes of Tumor Destruction. Photodiagnosis Photodyn. Ther..

[103] Palumbo G. (2007). Photodynamic Therapy and Cancer: A Brief Sightseeing Tour. Expert Opin. Drug Deliv..

[104] Kudinova N.V., Berezov T.T. (2010). Photodynamic Therapy of Cancer: Search for Ideal Photosensitizer. Biochem. Suppl. Ser. B Biomed. Chem..

[105] Anand S., Ortel B.J., Pereira S.P., Hasan T., Maytin E.V. (2012). Biomodulatory Approaches to Photodynamic Therapy for Solid Tumors. Cancer Lett..

[106] Stockert J.C., Cañete M., Juarranz A., Villanueva A., Horobin R.W., Borrell J.I., Teixidó J., Nonell S. (2007). Porphycenes: Facts and Prospects in Photodynamic Therapy of Cancer. Curr. Med. Chem..

[107] Lehmann P. (2007). Methyl Aminolaevulinate-Photodynamic Therapy: A Review of Clinical Trials in the Treatment of Actinic Keratoses and Nonmelanoma Skin Cancer. Br. J. Dermatol..

[108] O’Connor A.E., Gallagher W.M., Byrne A.T. (2009). Porphyrin and Nonporphyrin Photosensitizers in Oncology: Preclinical and Clinical Advances in Photodynamic Therapy. Photochem. Photobiol..

[109] Allison R.R., Sibata C.H. (2010). Oncologic Photodynamic Therapy Photosensitizers: A Clinical Review. Photodiagnosis Photodyn. Ther..

[110] Wachowska M., Muchowicz A., Firczuk M., Gabrysiak M., Winiarska M., Wańczyk M., Bojarczuk K., Golab J. (2011). Aminolevulinic Acid (Ala) as a Prodrug in Photodynamic Therapy of Cancer. Molecules.

[111] Zeitouni N.C., Paquette A.D., Housel J.P., Shi Y., Wilding G.E., Foster T.H., Henderson B.W. (2013). A Retrospective Review of Pain Control by a Two-Step Irradiance Schedule during Topical ALA-Photodynamic Therapy of Non-Melanoma Skin Cancer. Lasers Surg. Med..

[112] Baskaran R., Lee J., Yang S.-G. (2018). Clinical Development of Photodynamic Agents and Therapeutic Applications. Biomater. Res..

[113] Díez Valle R., Hadjipanayis C.G., Stummer W. (2019). Established and Emerging Uses of 5-ALA in the Brain: An Overview. J. Neurooncol..

[114] Santos K.L.M., Barros R.M., da Silva Lima D.P., Nunes A.M.A., Sato M.R., Faccio R., de Lima Damasceno B.P.G., Oshiro-Junior J.A. (2020). Prospective Application of Phthalocyanines in the Photodynamic Therapy against Microorganisms and Tumor Cells: A Mini-Review. Photodiagnosis Photodyn. Ther..

[115] Li X., De Zheng B., Peng X.H., Li S.Z., Ying J.W., Zhao Y., Huang J.D., Yoon J. (2019). Phthalocyanines as Medicinal Photosensitizers: Developments in the Last Five Years. Coord. Chem. Rev..

[116] Dąbrowski J.M., Pucelik B., Regiel-Futyra A., Brindell M., Mazuryk O., Kyzioł A., Stochel G., Macyk W., Arnaut L.G. (2016). Engineering of Relevant Photodynamic Processes through Structural Modifications of Metallotetrapyrrolic Photosensitizers. Coord. Chem. Rev..

[117] Yuan A., Wu J., Tang X., Zhao L., Xu F., Hu Y. (2013). Application of Near-Infrared Dyes for Tumor Imaging, Photothermal, and Photodynamic Therapies. J. Pharm. Sci..

[118] Shi C., Wu J.B., Pan D. (2016). Review on Near-Infrared Heptamethine Cyanine Dyes as Theranostic Agents for Tumor Imaging, Targeting, and Photodynamic Therapy. J. Biomed. Opt..

[119] Liu J., Zhang C., Rees T.W., Ke L., Ji L., Chao H. (2018). Harnessing Ruthenium(II) as Photodynamic Agents: Encouraging Advances in Cancer Therapy. Coord. Chem. Rev..

[120] Monro S., Colón K.L., Yin H., Roque J., Konda P., Gujar S., Thummel R.P., Lilge L., Cameron C.G., McFarland S.A. (2019). Transition Metal Complexes and Photodynamic Therapy from a Tumor-Centered Approach: Challenges, Opportunities, and Highlights from the Development of TLD1433. Chem. Rev..

[121] Chinna Ayya Swamy P., Sivaraman G., Priyanka R.N., Raja S.O., Ponnuvel K., Shanmugpriya J., Gulyani A. (2020). Near Infrared (NIR) Absorbing Dyes as Promising Photosensitizer for Photodynamic Therapy. Coord. Chem. Rev..

[122] Lo P.-C., Rodríguez-Morgade M.S., Pandey R.K., Ng D.K.P., Torres T., Dumoulin F. (2020). The Unique Features and Promises of Phthalocyanines as Advanced Photosensitisers for Photodynamic Therapy of Cancer. Chem. Soc. Rev..

[123] Pucelik B., Sułek A., Dąbrowski J.M. (2020). Bacteriochlorins and Their Metal Complexes as NIR-Absorbing Photosensitizers: Properties, Mechanisms, and Applications. Coord. Chem. Rev..

[124] Mfouo-Tynga I.S., Dias L.D., Inada N.M., Kurachi C. (2021). Features of Third Generation Photosensitizers Used in Anticancer Photodynamic Therapy: Review. Photodiagnosis Photodyn. Ther..

[125] Gomez S., Tsung A., Hu Z. (2020). Current Targets and Bioconjugation Strategies in Photodynamic Diagnosis and Therapy of Cancer. Molecules.

[126] Pereira P.M.R., Korsak B., Sarmento B., Schneider R.J., Fernandes R., Tomé J.P.C. (2015). Antibodies Armed with Photosensitizers: From Chemical Synthesis to Photobiological Applications. Org. Biomol. Chem..

[127] Fernandes S.R.G., Fernandes R., Sarmento B., Pereira P.M.R., Tomé J.P.C. (2019). Photoimmunoconjugates: Novel Synthetic Strategies to Target and Treat Cancer by Photodynamic Therapy. Org. Biomol. Chem..

[128] Sandland J., Boyle R.W. (2019). Photosensitizer Antibody-Drug Conjugates: Past, Present, and Future. Bioconjug. Chem..

[129] Mesquita M.Q., Dias C.J., Gamelas S., Fardilha M., Neves M.G.P.M.S., Faustino M.A.F. (2018). An Insight on the Role of Photosensitizer Nanocarriers for Photodynamic Therapy. An. Acad. Bras. Cienc..

[130] Oba T. (2007). Photosensitizer Nanoparticles for Photodynamic Therapy. Curr. Bioact. Compd..

[131] Juzenas P., Chen W., Sun Y.-P., Coelho M.A.N., Generalov R., Generalova N., Christensen I.L. (2008). Quantum Dots and Nanoparticles for Photodynamic and Radiation Therapies of Cancer. Adv. Drug Deliv. Rev..

[132] Chen W. (2008). Nanoparticle Self-Lighting Photodynamic Therapy for Cancer Treatment. J. Biomed. Nanotechnol..

[133] Li W.-T. (2010). Nanotechology-Based Strategies to Enhance the Efficacy of Photodynamic Therapy for Cancers. Curr. Drug Metab..

[134] Zhou L., Wei S., Ge X., Zhou J., Yu B., Shen J. (2012). External Heavy-Atomic Construction of Photosensitizer Nanoparticles for Enhanced in Vitro Photodynamic Therapy of Cancer. J. Phys. Chem. B.

[135] Lim C.K., Heo J., Shin S., Jeong K., Seo Y.H., Jang W.D., Park C.R., Park S.Y., Kim S., Kwon I.C. (2013). Nanophotosensitizers toward Advanced Photodynamic Therapy of Cancer. Cancer Lett..

[136] Debele T.A., Peng S., Tsai H.C. (2015). Drug Carrier for Photodynamic Cancer Therapy. Int. J. Mol. Sci..

[137] Shen Y., Shuhendler A.J., Ye D., Xu J.J., Chen H.Y. (2016). Two-Photon Excitation Nanoparticles for Photodynamic Therapy. Chem. Soc. Rev..

[138] Calixto G.M.F., Bernegossi J., De Freitas L.M., Fontana C.R., Chorilli M., Grumezescu A.M. (2016). Nanotechnology-Based Drug Delivery Systems for Photodynamic Therapy of Cancer: A Review. Molecules.

[139] Hong E.J., Choi D.G., Shim M.S. (2016). Targeted and Effective Photodynamic Therapy for Cancer Using Functionalized Nanomaterials. Acta Pharm. Sin. B.

[140] Li X., Kolemen S., Yoon J., Akkaya E.U. (2017). Activatable Photosensitizers: Agents for Selective Photodynamic Therapy. Adv. Funct. Mater..

[141] Abrahamse H., Kruger C.A., Kadanyo S., Mishra A. (2017). Nanoparticles for Advanced Photodynamic Therapy of Cancer. Photomed. Laser Surg..

[142] Chizenga E.P., Abrahamse H. (2020). Nanotechnology in Modern Photodynamic Therapy of Cancer: A Review of Cellular Resistance Patterns Affecting the Therapeutic Response. Pharmaceutics.

[143] Molaei M.J. (2020). Two-Dimensional (2D) Materials beyond Graphene in Cancer Drug Delivery, Photothermal and Photodynamic Therapy, Recent Advances and Challenges Ahead: A Review. J. Drug Deliv. Sci. Technol..

[144] Zhang C., Chen W., Zhang T., Jiang X., Hu Y. (2020). Hybrid Nanoparticle Composites Applied to Photodynamic Therapy: Strategies and Applications. J. Mater. Chem. B.

[145] Sztandera K., Gorzkiewicz M., Klajnert-Maculewicz B. (2020). Nanocarriers in Photodynamic Therapy—In Vitro and In Vivo Studies. Wiley Interdiscip. Rev. Nanomed. Nanobiotechnol..

[146] Shibu E.S., Hamada M., Murase N., Biju V. (2013). Nanomaterials Formulations for Photothermal and Photodynamic Therapy of Cancer. J. Photochem. Photobiol. C Photochem. Rev..

[147] Bugaj A.M. (2011). Targeted Photodynamic Therapy–A Promising Strategy of Tumor Treatment. Photochem. Photobiol. Sci..

[148] Lin L., Xiong L., Wen Y., Lei S., Deng X., Liu Z., Chen W., Miao X. (2015). Active Targeting of Nano-Photosensitizer Delivery Systems for Photodynamic Therapy of Cancer Stem Cells. J. Biomed. Nanotechnol..

[149] Weijer R., Broekgaarden M., Kos M., van Vught R., Rauws E.A.J., Breukink E., van Gulik T.M., Storm G., Heger M. (2015). Enhancing Photodynamic Therapy of Refractory Solid Cancers: Combining Second-Generation Photosensitizers with Multi-Targeted Liposomal Delivery. J. Photochem. Photobiol. C Photochem. Rev..

[150] Šošić L., Selbo P.K., Kotkowska Z.K., Kündig T.M., Høgset A., Johansen P. (2020). Photochemical Internalization: Light Paves Way for New Cancer Chemotherapies and Vaccines. Cancers.

[151] Zhou Z., Song J., Nie L., Chen X. (2016). Reactive Oxygen Species Generating Systems Meeting Challenges of Photodynamic Cancer Therapy. Chem. Soc. Rev..

[152] Kessel D. (2019). Apoptosis, Paraptosis and Autophagy: Death and Survival Pathways Associated with Photodynamic Therapy. Photochem. Photobiol..

[153] Nowis D., Makowski M., Stokłosa T., Legat M., Issat T., Goła̧b J. (2005). Direct Tumor Damage Mechanisms of Photodynamic Therapy. Acta Biochim. Pol..

[154] Plaetzer K., Kiesslich T., Oberdanner C.B., Krammer B. (2005). Apoptosis Following Photodynamic Tumor Therapy: Induction, Mechanisms and Detection. Curr. Pharm. Des..

[155] Castano A.P., Demidova T.N., Hamblin M.R. (2005). Mechanisms in Photodynamic Therapy: Part Two–Cellular Signaling, Cell Metabolism and Modes of Cell Death. Photodiagnosis Photodyn. Ther..

[156] Robertson C.A., Evans D.H., Abrahamse H. (2009). Photodynamic Therapy (PDT): A Short Review on Cellular Mechanisms and Cancer Research Applications for PDT. J. Photochem. Photobiol. B Biol..

[157] Mroz P., Yaroslavsky A., Kharkwal G.B., Hamblin M.R. (2011). Cell Death Pathways in Photodynamic Therapy of Cancer. Cancers.

[158] Kessel D., Oleinick N.L. (2018). Cell Death Pathways Associated with Photodynamic Therapy: An Update. Photochem. Photobiol..

[159] Kessel D. (2020). Photodynamic Therapy: Apoptosis, Paraptosis and Beyond. Apoptosis.

[160] Yoo J.O., Ha K.S. (2012). New Insights into the Mechanisms for Photodynamic Therapy-Induced Cancer Cell Death. Int. Rev. Cell Mol. Biol..

[161] Kushibiki T., Hirasawa T., Okawa S., Ishihara M. (2013). Responses of Cancer Cells Induced by Photodynamic Therapy. J. Healthc. Eng..

[162] Allison R.R., Moghissi K. (2013). Photodynamic Therapy (PDT): PDT Mechanisms. Clin. Endosc..

[163] Milla Sanabria L., Rodríguez M.E., Cogno I.S., Rumie Vittar N.B., Pansa M.F., Lamberti M.J., Rivarola V.A. (2013). Direct and Indirect Photodynamic Therapy Effects on the Cellular and Molecular Components of the Tumor Microenvironment. Biochim. Biophys. Acta-Rev. Cancer.

[164] Hwang H.S., Shin H., Han J., Na K. (2018). Combination of Photodynamic Therapy (PDT) and Anti-Tumor Immunity in Cancer Therapy. J. Pharm. Investig..

[165] Garg A.D., Agostinis P. (2014). ER Stress, Autophagy and Immunogenic Cell Death in Photodynamic Therapy-Induced Anti-Cancer Immune Responses. Photochem. Photobiol. Sci..

[166] Zawacka-Pankau J., Krachulec J., Grulkowski I., Bielawski K.P., Selivanova G. (2008). The P53-Mediated Cytotoxicity of Photodynamic Therapy of Cancer: Recent Advances. Toxicol. Appl. Pharmacol..

[167] Bhuvaneswari R., Gan Y.Y., Soo K.C., Olivo M. (2009). The Effect of Photodynamic Therapy on Tumor Angiogenesis. Cell. Mol. Life Sci..

[168] Broekgaarden M., Weijer R., van Gulik T.M., Hamblin M.R., Heger M. (2015). Tumor Cell Survival Pathways Activated by Photodynamic Therapy: A Molecular Basis for Pharmacological Inhibition Strategies. Cancer Metastasis Rev..

[169] Xu J., Gao J., Wei Q. (2016). Combination of Photodynamic Therapy with Radiotherapy for Cancer Treatment. J. Nanomater..

[170] Castano A.P., Mroz P., Hamblin M.R. (2006). Photodynamic Therapy and Anti-Tumour Immunity. Nat. Rev. Cancer.

[171] Garg A.D., Nowis D., Golab J., Agostinis P. (2010). Photodynamic Therapy: Illuminating the Road from Cell Death towards Anti-Tumour Immunity. Apoptosis.

[172] Mroz P., Hashmi J.T., Huang Y.-Y., Lange N., Hamblin M.R. (2011). Stimulation of Anti-Tumor Immunity by Photodynamic Therapy. Expert Rev. Clin. Immunol..

[173] Garg A.D., Krysko D.V., Vandenabeele P., Agostinis P. (2011). DAMPs and PDT-Mediated Photo-Oxidative Stress: Exploring the Unknown. Photochem. Photobiol. Sci..

[174] Rodríguez M.E., Cogno I.S., Milla Sanabria L.S., Morán Y.S., Rivarola V.A. (2016). Heat Shock Proteins in the Context of Photodynamic Therapy: Autophagy, Apoptosis and Immunogenic Cell Death. Photochem. Photobiol. Sci..

[175] Nath S., Obaid G., Hasan T. (2019). The Course of Immune Stimulation by Photodynamic Therapy: Bridging Fundamentals of Photochemically Induced Immunogenic Cell Death to the Enrichment of T-Cell Repertoire. Photochem. Photobiol..

[176] Asadzadeh Z., Safarzadeh E., Safaei S., Baradaran A., Mohammadi A., Hajiasgharzadeh K., Derakhshani A., Argentiero A., Silvestris N., Baradaran B. (2020). Current Approaches for Combination Therapy of Cancer: The Role of Immunogenic Cell Death. Cancers.

[177] Beltrán Hernández I., Yu Y., Ossendorp F., Korbelik M., Oliveira S. (2020). Preclinical and Clinical Evidence of Immune Responses Triggered in Oncologic Photodynamic Therapy: Clinical Recommendations. J. Clin. Med..

[178] Brackett C.M., Gollnick S.O. (2011). Photodynamic Therapy Enhancement of Anti-Tumor Immunity. Photochem. Photobiol. Sci..

[179] Panzarini E., Inguscio V., Dini L. (2013). Immunogenic Cell Death: Can It Be Exploited in Photodynamic Therapy for Cancer?. Biomed. Res. Int..

[180] Anzengruber F., Avci P., De Freitas L.F., Hamblin M.R. (2015). T-Cell Mediated Anti-Tumor Immunity after Photodynamic Therapy: Why Does It Not Always Work and How Can We Improve It?. Photochem. Photobiol. Sci..

[181] Alzeibak R., Mishchenko T.A., Shilyagina N.Y., Balalaeva I.V., Vedunova M.V., Krysko D.V. (2021). Targeting Immunogenic Cancer Cell Death by Photodynamic Therapy: Past, Present and Future. J. Immunother. Cancer.

[182] Dang J., He H., Chen D., Yin L. (2017). Manipulating Tumor Hypoxia toward Enhanced Photodynamic Therapy (PDT). Biomater. Sci..

[183] Li X., Kwon N., Guo T., Liu Z., Yoon J. (2018). Innovative Strategies for Hypoxic-Tumor Photodynamic Therapy. Angew. Chem. Int. Ed..

[184] Pucelik B., Sułek A., Barzowska A., Dąbrowski J.M. (2020). Recent Advances in Strategies for Overcoming Hypoxia in Photodynamic Therapy of Cancer. Cancer Lett..

[185] Wei F., Rees T.W., Liao X., Ji L., Chao H. (2021). Oxygen Self-Sufficient Photodynamic Therapy. Coord. Chem. Rev..

[186] Hu T., Wang Z., Shen W., Liang R., Yan D., Wei M. (2021). Recent Advances in Innovative Strategies for Enhanced Cancer Photodynamic Therapy. Theranostics.

[187] De Vijlder H.C., Sterenborg H.J.C.M., Martino Neumann H.A., Robinson D.J., De Haas E.R.M. (2012). Light Fractionation Significantly Improves the Response of Superficial Basal Cell Carcinoma to Aminolaevulinic Acid Photodynamic Therapy: Five-Year Follow-up of a Randomized, Prospective Trial. Acta Derm. Venereol..

[188] Shams M., Owczarczak B., Manderscheid-Kern P., Bellnier D.A., Gollnick S.O. (2015). Development of Photodynamic Therapy Regimens That Control Primary Tumor Growth and Inhibit Secondary Disease. Cancer Immunol. Immunother..

[189] Zuluaga M.-F., Lange N. (2008). Combination of Photodynamic Therapy with Anti-Cancer Agents. Curr. Med. Chem..

[190] Dąbrowski J.M., Arnaut L.G. (2015). Photodynamic Therapy (PDT) of Cancer: From Local to Systemic Treatment. Photochem. Photobiol. Sci..

[191] Wilson B.C., Patterson M.S., Lilge L. (1997). Implicit and Explicit Dosimetry in Photodynamic Therapy: A New Paradigm. Lasers Med. Sci..

[192] Zhu T.C., Finlay J.C., Wilson B. (2005). TH-A-T-6C-01: Photodynamic Therapy: Fundamentals and Dosimetry. Med. Phys..

[193] Kim M.M., Darafsheh A. (2020). Light Sources and Dosimetry Techniques for Photodynamic Therapy. Photochem. Photobiol..

[194] Pogue B.W., Elliott J.T., Kanick S.C., Davis S.C., Samkoe K.S., Maytin E.V., Pereira S.P., Hasan T. (2016). Revisiting Photodynamic Therapy Dosimetry: Reductionist & Surrogate Approaches to Facilitate Clinical Success. Phys. Med. Biol..

[195] Jacques S.L. (2010). How Tissue Optics Affect Dosimetry of Photodynamic Therapy. J. Biomed. Opt..

[196] Swartling J., Axelsson J., Ahlgren G., Kälkner K.M., Nilsson S., Svanberg S., Svanberg K., Andersson-Engels S. (2010). System for Interstitial Photodynamic Therapy with Online Dosimetry: First Clinical Experiences of Prostate Cancer. J. Biomed. Opt..

[197] Li B., Lin L., Lin H., Wilson B.C. (2016). Photosensitized Singlet Oxygen Generation and Detection: Recent Advances and Future Perspectives in Cancer Photodynamic Therapy. J. Biophotonics.

[198] Kim M.M., Ghogare A.A., Greer A., Zhu T.C. (2017). On the in Vivo Photochemical Rate Parameters for PDT Reactive Oxygen Species Modeling. Phys. Med. Biol..

[199] Mallidi S., Spring B.Q., Chang S., Vakoc B., Hasan T. (2015). Optical Imaging, Photodynamic Therapy and Optically Triggered Combination Treatments. Cancer J..

[200] Celli J.P., Spring B.Q., Rizvi I., Evans C.L., Samkoe K.S., Verma S., Pogue B.W., Hasan T. (2010). Imaging and Photodynamic Therapy: Mechanisms, Monitoring, and Optimization. Chem. Rev..

[201] De Silva P., Saad M.A., Thomsen H.C., Bano S., Ashraf S., Hasan T. (2020). Photodynamic Therapy, Priming and Optical Imaging: Potential Co-Conspirators in Treatment Design and Optimization–A Thomas Dougherty Award for Excellence in PDT Paper. J. Porphyr. Phthalocyanines.

[202] Hamblin M.R., Huang Y. (2017). Imaging in Photodynamic Therapy.

[203] Hester S.C., Kuriakose M., Nguyen C.D., Mallidi S. (2020). Role of Ultrasound and Photoacoustic Imaging in Photodynamic Therapy for Cancer. Photochem. Photobiol..

[204] Kachynski A.V., Pliss A., Kuzmin A.N., Ohulchanskyy T.Y., Baev A., Qu J., Prasad P.N. (2014). Photodynamic Therapy by in Situ Nonlinear Photon Conversion. Nat. Photonics.

[205] Parodi V., Jacchetti E., Osellame R., Cerullo G., Polli D., Raimondi M.T. (2020). Nonlinear Optical Microscopy: From Fundamentals to Applications in Live Bioimaging. Front. Bioeng. Biotechnol..

[206] Brancaleon L., Moseley H. (2002). Laser and Non-Laser Light Sources for Photodynamic Therapy. Lasers Med. Sci..

[207] Mang T.S. (2004). Lasers and Light Sources for PDT: Past, Present and Future. Photodiagnosis Photodyn. Ther..

[208] Huang Z., Xu H., Meyers A.D., Musani A.I., Wang L., Tagg R., Barqawi A.B., Chen Y.K. (2008). Photodynamic Therapy for Treatment of Solid Tumors–Potential and Technical Challenges. Technol. Cancer Res. Treat..

[209] Finlay J.C., Darafsheh A., Wong B.J.-F., Ilgner J. (2016). Light sources, drugs, and dosimetry. Biomedical Optics in Otorhinolaryngology: Head and Neck Surgery.

[210] Mallidi S., Anbil S., Bulin A.L., Obaid G., Ichikawa M., Hasan T. (2016). Beyond the Barriers of Light Penetration: Strategies, Perspectives and Possibilities for Photodynamic Therapy. Theranostics.

[211] Hu J., Tang Y., Elmenoufy A.H., Xu H., Cheng Z., Yang X. (2015). Nanocomposite-Based Photodynamic Therapy Strategies for Deep Tumor Treatment. Small.

[212] Magalhães C.M., Esteves da Silva J.C.G., Pinto da Silva L. (2016). Chemiluminescence and Bioluminescence as an Excitation Source in the Photodynamic Therapy of Cancer: A Critical Review. ChemPhysChem.

[213] Fan W., Huang P., Chen X. (2016). Overcoming the Achilles’ Heel of Photodynamic Therapy. Chem. Soc. Rev..

[214] Wang C., Cheng L., Liu Z. (2013). Upconversion Nanoparticles for Photodynamic Therapy and Other Cancer Therapeutics. Theranostics.

[215] Hamblin M.R. (2018). Upconversion in Photodynamic Therapy: Plumbing the Depths. Dalt. Trans..

[216] Liu Y., Meng X., Bu W. (2019). Upconversion-Based Photodynamic Cancer Therapy.

[217] Cline B., Delahunty I., Xie J. (2019). Nanoparticles to Mediate X-Ray-Induced Photodynamic Therapy and Cherenkov Radiation Photodynamic Therapy. Wiley Interdiscip. Rev. Nanomed. Nanobiotechnol..

[218] Chen X., Song J., Chen X., Yang H. (2019). X-Ray-Activated Nanosystems for Theranostic Applications. Chem. Soc. Rev..

[219] Sun W., Zhou Z., Pratx G., Chen X., Chen H. (2020). Nanoscintillator-Mediated X-Ray Induced Photodynamic Therapy for Deep-Seated Tumors: From Concept to Biomedical Applications. Theranostics.

[220] Ren X.D., Hao X.Y., Li H.C., Ke M.R., Zheng B.Y., Huang J.D. (2018). Progress in the Development of Nanosensitizers for X-Ray-Induced Photodynamic Therapy. Drug Discov. Today.

[221] Larue L., Ben Mihoub A., Youssef Z., Colombeau L., Acherar S., André J.C., Arnoux P., Baros F., Vermandel M., Frochot C. (2018). Using X-Rays in Photodynamic Therapy: An Overview. Photochem. Photobiol. Sci..

[222] Daouk J., Dhaini B., Petit J., Frochot C., Barberi-Heyob M., Schohn H. (2020). Can Cerenkov Light Really Induce an Effective Photodynamic Therapy?. Radiation.

[223] Huang Y., Qiu F., Chen R., Yan D., Zhu X. (2020). Fluorescence Resonance Energy Transfer-Based Drug Delivery Systems for Enhanced Photodynamic Therapy. J. Mater. Chem. B.

[224] Fan W., Lu N., Xu C., Liu Y., Lin J., Wang S., Shen Z., Yang Z., Qu J., Wang T. (2017). Enhanced Afterglow Performance of Persistent Luminescence Implants for Efficient Repeatable Photodynamic Therapy. ACS Nano.

[225] Hu L., Wang P., Zhao M., Liu L., Zhou L., Li B., Albaqami F.H., El-Toni A.M., Li X., Xie Y. (2018). Near-Infrared Rechargeable “Optical Battery” Implant for Irradiation-Free Photodynamic Therapy. Biomaterials.

[226] Teh D.B.L., Bansal A., Chai C., Toh T.B., Tucker R.A.J., Gammad G.G.L., Yeo Y., Lei Z., Zheng X., Yang F. (2020). A Flexi-PEGDA Upconversion Implant for Wireless Brain Photodynamic Therapy. Adv. Mater..

[227] Bansal A., Yang F., Xi T., Zhang Y., Ho J.S. (2018). In Vivo Wireless Photonic Photodynamic Therapy. Proc. Natl. Acad. Sci. USA.

[228] Yamagishi K., Kirino I., Takahashi I., Amano H., Takeoka S., Morimoto Y., Fujie T. (2019). Tissue-Adhesive Wirelessly Powered Optoelectronic Device for Metronomic Photodynamic Cancer Therapy. Nat. Biomed. Eng..

[229] Kim A., Zhou J., Samaddar S., Song S.H., Elzey B.D., Thompson D.H., Ziaie B. (2019). An Implantable Ultrasonically-Powered Micro-Light-Source (ΜLight) for Photodynamic Therapy. Sci. Rep..

[230] Algorri J.F., Ochoa M., Roldán-Varona P., Rodríguez-Cobo L., López-Higuera J.M. (2021). Light Technology for Efficient and Effective Photodynamic Therapy: A Critical Review. Cancers.

