# Supplementary material for: Photodynamic Therapy: A Compendium of Latest Reviews
Source: Cancers (Basel). 2021 Sep 3;13(17):4447. doi: 10.3390/cancers13174447 (PMC8430498; doi:10.3390/cancers13174447)
Supplement: Supplementary file 1 [file cancers-13-04447-s001.zip › cancers-1349074-supplementary.pdf]

Table S1. Main characteristics of some common photosensitizers.

porphyrin-based photosensitizers

PS/<br>Drug (substance)	Lambda (nm)	Time to PDT after dose delivery	Clearance	Clinical/preclinical application [1]	Clinical approved?	Side effects [1]	Localization	Primary mechanism of action	Manufacturer [2]	Refs.
Photofrin®<br>1st generation	630	24-48 h	4-12 weeks [3]	**Approved**<br>Esophageal cancer, lung cancer, microinvasive endobronchial cancer, gastric and papillary bladder, cervical dysplasia and cancer.	Yes Worldwide	Mild to moderate erythema Photosensiticity, mild constipation	Mitochondrial membrane and lysosome [4]<br>Golgi apparatus [1]	Ischemic cell necrosis[1]<br>Rapid illumination post infusion will favor vascular shutdown [5]	Axcan harma, Canada	[3]<br>[6]
TOOKAD®<br>(pheophorbides)	753	15 min	24-48h [7]	**Clinical trial:** Prostate Cancer.	EMA Yes<br>FDA No	-	Vascular occlusion [8]	Vascular damage [1]	Negma-Lerads	[7]
Foscan®<br>(Temoporfin)	652	48-96 h	Dark 1 day<br>1-7 home [9]	**Preclinical testing:**<br>Pancreas, Breast<br>**Approved:**<br>Head and neck Cancer.	EMA, Norway, Iceland	Swelling, bleeding, ulceration, scarring Photosensitivity	Mitochondria [10]<br>Endoplasmic reticulum (ER) [1]	Vascular damage and direct tumor cytotoxicity [1]	Biolitec, Germany	[11]<br>[12]<br>[13]
Purlytin® (purpirins)	664	24 h	7-14 days [14]	**Clinical trials:**<br>Metastatic breast cancer, AIDS related Kaposis sarcoma, basal cell carcinomas	No	Photosensitivity	Accumulates in cellular membranes [5]<br>Mitochondria, lysosomes [1]	Direct tumor cytotoxicity [1]		[14]
Lutrin® and Lutex® (metalloporphyrins) [1]	732	3h		**Clinical Trials:**<br>Recurrent prostate cancer, cervical cancer<br>**Preclinical testing:**<br>Recurrent breast cancer	No	Photosensitivity (minimal)	Lysosome	Vascular damage and direct tumor cytotoxicity		[15]
Laserphyrin® / NPe6 / Talaporfin (Chlorin m-THPC) [1]	664	2-4h	Dark 2 days<br>Fluorescence 2-5days<br>Home 8-14 days [16]	**Approved (Japan):**<br>Early lung cancer<br>**Clinical trials**<br>Hepatocellular cancer, Liver metastasis	Japan		Lysosome, endosome	Vascular stasis and direct tumor cytotoxicity	Meiji Seika, Japan	[16]
Levulan®<br>(Protoporphyrin) [1]	635	4-6h	48hours [17]	**Approved:**<br>Actinic keratoses	EMA, USA, Austria, China	Stinging, burning, itching, erythema	Mitochondria, cytosol, cytosolic membranes	Direct tumor cytotoxicity	DUSA, USA	[18]
Metvix® / Metvixia®<br>(Protoporphyrin) [1]	635	3h	48h	**Approved:**<br>Actinic keratoses, basal cell carcinoma	EMA, USA, Canada	Burning sensation, redness, scabbing	Mitochondria, cytosol, cytosolic membranes	Direct tumor cytotoxicity	Galderma, UK	[19]
Redaporfin® [2]	749	24-72h [20]		**Clinical trials:**<br>Biliary tract cancer<br>Head neck	No		Endoplasmic reticulum and Golgi apparatus.[21]	Vascular [20]	Luzitin, Portugal	[22]<br>[23]
Visudyne®<br>(Verteporfin)	690	1 h		**Approved:**<br>Age-related macular degeneration	Norway, China		DNA Fragmentation [10]<br>Mitochondria membrane [24]		Novartis, Switzerland	[25]<br>[26]
Ameluz®/Levulan®<br>(5-ALA)	635	3h	48h	**Approved:**<br>Actinic Keratosis<br>**Clinical trials:**<br>Brain	Yes	Mild to moderate actinic keratosis			DUSA, USA	[27]
Photochlor (HPPH)	655			BCC, lung, head and neck cancers	USA		Selectively accumulates in the cytoplasm of cancer or pre-cancerous cells		Roswell Park Cancer Institute	[28]

Other photosensitizers

Hypericin [1]	595	0.5-6h		**Preclinical testing:** Bladder cancer, nasopharyngeal cancer	No	Slight damage to surrounding normal tissue	Membranes of endoplasmic reticulum (ER), membranes of Golgi	Tumor cell cytotoxicity and vascular damage		
Chalcogenopyrylium Dyes [1]	592-746	24h		**Preclinical testing:** Prostate cancer, breast cancer, colon cancer	No	Dark toxicity	Lysosomes	Tumor cell and cytotoxicity		
Phenothiazinium dye—methylene Blue [1]	666	1-4h		**Preclinical testing:** Bladder cancer, colon cancer, AIDS-related Kaposi's sarcoma	No		Lysosomes	Tumor cell and cytotoxicity		
Phenothiazinium dye—Nile blue and derivatives [1]	626,632	1h		**Preclinical testing:** AIDS-related Kaposi's sarcoma, T-cell leukemia	No	Minimal side effects	ER, Golgi apparatus	Tumor cell and cytotoxicity		
Phenothiazinium dye—toluidine Blue [1]	650-660	30min		**Preclinical testing:** Bladder cancer, mouse mammary sarcoma	No	Minimal side effects	Lysosomes	Tumor cell and cytotoxicity		
Cyanines [1]	535-574	1h		**Preclinical testing:** Leukemia, lymphoma	No	Minimal side effects	Mitochondria, plasma membrane	Tumor cell and cytotoxicity		
ADPM06 [1]	680	<5min		**Preclinical testing:** Breast cancer, mouse lung cancer	No		ER, mitochondria	Vascular-targeted		
Zinc phthalocyanine 2nd	675	12 h			No		These PS accumulate in mitochondria and apparently induces apoptosis [5]			[29]
Motexafin lutetium (Lutex)	720	3-24 h			No		Lutex binds to lysosomes of EMT6 cells in vitro and to produce apoptosis [10]			[30]
Mono-L-aspartyl chlorin	664-667	4 h	3weeks [31]		No		Increased expression of the mRNA of IL-2, IL-6, and TNF-α 6 h later [10]			[31]
Phenotiazonium salt, MB	640	24h			No					[32]

References:

1. O'Connor, A.E.; Gallagher, W.M.; Byrne, A.T. Porphyrin and nonporphyrin photosensitizers in oncology: Preclinical and clinical advances in photodynamic therapy. *Photochem. Photobiol.* 2009, *85*, 1053–1074.
2. Baskaran, R.; Lee, J.; Yang, S.-G. Clinical development of photodynamic agents and therapeutic applications. *Biomater. Res.* **2018**, *22*, 25, doi:10.1186/s40824-018-0140-z.
3. Khan, S.A.; Dougherty, T.J.; Mang, T.S. An evaluation of photodynamic therapy in the management of cutaneous metastases of breast cancer. *Eur. J. Cancer* **1993**, *29A*, 1686–90, doi:10.1016/0959-8049(93)90105-o.
4. Kessel, D. Photodynamic therapy: Promotion of efficacy by a sequential protocol. *J. Porphyr. Phthalocyanines* **2016**, *20*, 302–306, doi:10.1142/S1088424616500073.
5. Allison, R.R.; Sibata, C.H. Oncologic photodynamic therapy photosensitizers: A clinical review. *Photodiagnosis Photodyn. Ther.* 2010, *7*, 61–75.
6. Ahn, T.G.; Lee, B.R.; Choi, E.Y.; Kim, D.W.; Han, S.J. Photodynamic therapy for breast cancer in a BALB/c mouse model. *J. Gynecol. Oncol.* **2012**, *23*, 115–119, doi:10.3802/jgo.2012.23.2.115.
7. Azzouzi, A.R.; Barret, E.; Moore, C.M.; Villers, A.; Allen, C.; Scherz, A.; Muir, G.; De Wildt, M.; Barber, N.J.; Lebdai, S.; et al. TOOKAD® Soluble vascular-targeted photodynamic (VTP) therapy: Determination of optimal treatment conditions and assessment of effects in patients with localised prostate cancer. *BJU Int.* **2013**, *112*, 766–774, doi:10.1111/bju.12265.
8. Banerjee, S.M.M.; MacRobert, A.J.J.; Mosse, C.A.A.; Periera, B.; Bown, S.G.G.; Keshtgar, M.R.S.R.S. *Photodynamic therapy: Inception to application in breast cancer*; Churchill Livingstone, 2017; Vol. 31, pp. 105–113;.
9. Biogen Inc ANNEX I SUMMARY OF PRODUCT CHARACTERISTICS Tecfidera 2013.

10. Castano, A.P.; Demidova, T.N.; Hamblin, M.R. Mechanisms in photodynamic therapy: Part two - Cellular signaling, cell metabolism and modes of cell death. *Photodiagnosis Photodyn. Ther.* 2005, *2*, 1–23.

11. Cuenca, R.E.; Allison, R.R.; Sibata, C.; Downie, G.H. Breast cancer with chest wall progression: treatment with photodynamic therapy. *Ann. Surg. Oncol.* **2004**, *11*, 322–7, doi:10.1245/aso.2004.03.025.

12. Wyss, P.; Schwarz, V.; Dobler-Girdziunaite, D.; Hornung, R.; Walt, H.; Degen, A.; Fehr, M. Photodynamic therapy of locoregional breast cancer recurrences using a chlorin-type photosensitizer. *Int. J. Cancer* **2001**, *93*, 720–724, doi:10.1002/ijc.1400.

13. Bown, S.G.; Hatfield, A.W.R.; Rogowska, A.Z.; Whitelaw, D.E.; Lees, W.R.; Lovat, L.B.; Ripley, P.; Jones, L.; Wyld, P.; Gillams, A. Photodynamic therapy for cancer of the pancreas. *Gut* **2002**, *50*, 549–557, doi:10.1136/gut.50.4.549.

14. Bonnett, R.; Martínez, G. Photobleaching of sensitisers used in photodynamic therapy. *Tetrahedron* 2001, *57*, 9513–9547.

15. Griffin, G.M.; Zhu, T.; Solonenko, M.; Del Piero, F.; Kapakin, A.; Busch, T.M.; Yodh, A.; Polin, G.; Bauer, T.; Fraker, D.; et al. Preclinical evaluation of motexafin lutetium-mediated intraperitoneal photodynamic therapy in a canine model. *Clin. Cancer Res.* **2001**, *7*, 374–81.

16. Nakamura, T.; Oinuma, T. Usefulness of photodynamic diagnosis and therapy using talaporfin sodium for an advanced-aged patient with inoperable gastric cancer (a secondary publication). *Laser Ther.* **2014**, *23*, 201–210, doi:10.5978/islsm.14-OR-16.

17. Ericson, M.B.; Wennberg, A.M.; Larkö, O. Review of photodynamic therapy in actinic keratosis and basal cell carcinoma. *Ther. Clin. Risk Manag.* 2008, *4*, 1–9.

18. Osiecka, B.J.; Jurczyszyn, K.; Ziółkowski, P. The application of Levulan®-based photodynamic therapy with imiquimod in the treatment of recurrent basal cell carcinoma. *Med. Sci. Monit.* **2012**, *18*, PI5-9, doi:10.12659/msm.882449.

19. Freeman, M.; Vinciullo, C.; Francis, D.; Spelman, L.; Nguyen, R.; Fergin, P.; Thai, K.E.; Murrell, D.; Weightman, W.; Anderson, C.; et al. A comparison of photodynamic therapy using topical methyl aminolevulinate (Metvix®) with single cycle cryotherapy in patients with actinic keratosis: A prospective, randomized study. *J. Dermatolog. Treat.* **2003**, *14*, 99–106, doi:10.1080/09546630310012118.

20. Rocha, L.G.B. Development of a novel photosensitizer for photodynamic therapy of cancer.

21. Gomes- da- Silva, L.C.; Zhao, L.; Bezu, L.; Zhou, H.; Sauvat, A.; Liu, P.; Durand, S.; Leduc, M.; Souquere, S.; Loos, F.; et al. Photodynamic therapy with redaporfin targets the endoplasmic reticulum and Golgi apparatus. *EMBO J.* **2018**, *37*, doi:10.15252/embj.201798354.

22. S. Lobo, A.C.; Gomes-da-Silva, L.C.; Rodrigues-Santos, P.; Cabrita, A.; Santos-Rosa, M.; Arnaut, L.G. Immune Responses after Vascular Photodynamic Therapy with Redaporfin. *J. Clin. Med.* **2019**, *9*, 104, doi:10.3390/jcm9010104.

23. Santos, L.L.; Oliveira, J.; Monteiro, E.; Santos, J.; Sarmento, C. Treatment of Head and Neck Cancer with Photodynamic Therapy with Redaporfin: A Clinical Case Report. *Case Rep. Oncol.* **2018**, *11*, 769–776, doi:10.1159/000493423.

24. Mahalingam, S.M.; Ordaz, J.D.; Low, P.S. Targeting of a Photosensitizer to the Mitochondrion Enhances the Potency of Photodynamic Therapy. *ACS Omega* **2018**, *3*, 6066–6074, doi:10.1021/acsomega.8b00692.

25. Detty, M.R.; Gibson, S.L.; Wagner, S.J. Current clinical and preclinical photosensitizers for use in photodynamic therapy. *J. Med. Chem.* 2004, *47*, 3897–3915.

26. Banerjee; El-Sheikh; Malhotra; Mosse; Parker; Williams; MacRobert; Hamoudi; Bown; Keshtgar Photodynamic Therapy in Primary Breast Cancer. *J. Clin. Med.* **2020**, *9*, 483, doi:10.3390/jcm9020483.

27. Díez Valle, R.; Hadjipanayis, C.G.; Stummer, W. Established and emerging uses of 5-ALA in the brain: an overview. *J. Neurooncol.* 2019, *141*, 487–494.

28. Q, X.; J, W.; X, P.; Y, J.; P, W.; AW, L.; L, G.; S, J.; C, X. Discovery and Development of Natural Products and their Derivatives as Photosensitizers for Photodynamic Therapy. *Curr. Med. Chem.* **2018**, *25*, 839–860, doi:10.2174/0929867324666170823143137.

29. Mfouo-Tynga, I.; Houreld, N.N.; Abrahamse, H. Induced Cell Death Pathway Post Photodynamic Therapy Using a Metallophthalocyanine Photosensitizer in Breast Cancer Cells. *Photomed. Laser Surg.* **2014**, *32*, 205–211, doi:10.1089/pho.2013.3650.

30. MacDonald, I.J.; Dougherty, T.J. Basic principles of photodynamic therapy. In Proceedings of the Journal of Porphyrins and Phthalocyanines; John Wiley and Sons Ltd, 2001; Vol. 5, pp. 105–129.

31. Taber, S.W.; Fingar, V.H.; Coots, C.T.; Wieman, T.J. Photodynamic therapy using mono-L-aspartyl chlorin e6 (Npe6) for the treatment of cutaneous disease: a Phase I clinical study. *Clin. Cancer Res.* **1998**, *4*, 2741–6.

32. dos Santos, A.F.; Terra, L.F.; Wailemann, R.A.M.; Oliveira, T.C.; de Morais Gomes, V.; Mineiro, M.F.; Meotti, F.C.; Bruni-Cardoso, A.; Baptista, M.S.; Labriola, L. Methylene blue photodynamic therapy induces selective and massive cell death in human breast cancer cells. *BMC Cancer* **2017**, *17*, 194, doi:10.1186/s12885-017-3179-7.
